# Somatostatin-Positive Neurons in the Rostral Zona Incerta Modulate Innate Fear-Induced Defensive Response in Mice

**DOI:** 10.1007/s12264-022-00958-y

**Published:** 2022-10-19

**Authors:** Shan Lin, Meng-Yue Zhu, Meng-Yu Tang, Mi Wang, Xiao-Dan Yu, Yi Zhu, Shi-Ze Xie, Dan Yang, Jiadong Chen, Xiao-Ming Li

**Affiliations:** 1 Department of Neurobiology and Department of Neurology of the Second Affiliated Hospital, Zhejiang University School of Medicine, Hangzhou, 310058 China; 2 NHC and CAMS Key Laboratory of Medical Neurobiology, MOE Frontier Science Center for Brain Research and Brain-Machine Integration, School of Brain Science and Brian Medicine, Zhejiang University, Hangzhou, 310058 China; 3 Center for Brain Science and Brain-Inspired Intelligence, Research Units for Emotion and Emotion Disorders, Chinese Academy of Medical Sciences, China/Guangdong-Hong Kong-Macao Greater Bay Area, Joint Institute for Genetics and Genome Medicine between Zhejiang University and the University of Toronto, Hangzhou, 310058 China

**Keywords:** Innate fear, Zona incerta, Somatostatin-positive neurons, Looming stimulus, Defensive behavior

## Abstract

Defensive behaviors induced by innate fear or Pavlovian fear conditioning are crucial for animals to avoid threats and ensure survival. The zona incerta (ZI) has been demonstrated to play important roles in fear learning and fear memory, as well as modulating auditory-induced innate defensive behavior. However, whether the neuronal subtypes in the ZI and specific circuits can mediate the innate fear response is largely unknown. Here, we found that somatostatin (SST)-positive neurons in the rostral ZI of mice were activated by a visual innate fear stimulus. Optogenetic inhibition of SST-positive neurons in the rostral ZI resulted in reduced flight responses to an overhead looming stimulus. Optogenetic activation of SST-positive neurons in the rostral ZI induced fear-like defensive behavior including increased immobility and bradycardia. In addition, we demonstrated that manipulation of the GABAergic projections from SST-positive neurons in the rostral ZI to the downstream nucleus reuniens (Re) mediated fear-like defensive behavior. Retrograde trans-synaptic tracing also revealed looming stimulus-activated neurons in the superior colliculus (SC) that projected to the Re-projecting SST-positive neurons in the rostral ZI (SC-ZIr^SST^-Re pathway). Together, our study elucidates the function of SST-positive neurons in the rostral ZI and the SC-ZIr^SST^-Re tri-synaptic circuit in mediating the innate fear response.

**Supplementary Information:**

The online version contains supplementary material available at 10.1007/s12264-022-00958-y.

## Introduction

The zona incerta (ZI), originally described as a “zone of uncertainty” [1], consists of heterogeneous neuron subtypes and extensive efferent and afferent projections connected with various regions across the brain [2]. Recent studies have revealed the involvement of the ZI in various functions including binge-like eating [3], hunting [4, 5], sleep [6], parkinsonian motor symptoms [7], fear learning and fear memory [8–11], anxiety [12], and novelty-seeking [13], as well as neuron development [14]. These behaviors are closely associated with physiological functions that are beneficial for the well-being and survival of animals [15].

Defensive responses induced by innate fear or Pavlovian fear conditioning are crucial for animal survival. The GABAergic neurons in the ZI form extensive synaptic connections with diverse fear-related brain regions, such as the amygdala [9], thalamus [11], and periaqueductal gray (PAG) [8]. Manipulating the GABAergic neuronal activity in the ZI regulates associative fear learning and fear memory. For example, GABAergic projections in the central nucleus of the amygdala to the parvalbumin (PV)-positive neurons in the ZI are required for fear memory acquisition and retrieval [9]. GABAergic neurons in the ZI can modulate fear generalization that is associated with trauma- and anxiety-related disorders [10]. In addition, both GABAergic neurons in the rostral ZI and PV-positive neurons in the ZI demonstrate gain modulation of the sound-induced innate flight response [8, 16]. However, whether the neuronal subtypes in the ZI can mediate the innate fear response remains to be explored.

Innate fear is an instinctive fear of threat that does not depend on the experience of an external harmful stimulus or on fear learning associating a valence of danger to a threat [17]. Defensive responses can be triggered by sensory stimuli from predators, such as olfactory, visual, or auditory cues. In the laboratory, a rapidly expanding overhead dark disk mimics the visual threat from a fast-approaching aerial predator. Such a looming stimulus can induce either freezing for an extended period or flight to a provided shelter [18]. Brain regions including the superior colliculus (SC) [19–22], ventral midline thalamus [23], and ventral lateral geniculate nucleus [24, 25] have been shown to regulate the looming-induced innate fear response. The ZI receives projections from the visual cortex and SC [4, 26], but whether and how the ZI participates in looming-induced defensive behavior is not known.

In the present study, we demonstrated that somatostatin (SST)-positive neurons in the rostral ZI of mice were activated by an overhead looming visual stimulus using *in vivo* fiber photometry. We found that optogenetic manipulation of SST-positive neurons in the rostral ZI attenuated the looming stimulus-induced defensive response. Surprisingly, optogenetic activation of these neurons induced fear-like defensive behaviors including increased immobility in the open field and bradycardia. We further revealed a tri-synaptic circuit from the SC to the Re-projecting SST-positive neurons in the rostral ZI that were crucial for regulating the looming stimulus-induced defensive behavior.

## Methods

### Animals

All experiments were conducted in accordance with the guidelines for the care and use of laboratory animals of Zhejiang University (ZJU, Hangzhou, China) and were approved by the Animal Advisory Committee at ZJU. SST-Cre (No. 013044) and Ai14 (No. 007908) mice were obtained from the Jackson Laboratory (Bar Harbor, USA) and bred in the animal facility at ZJU. All mice were housed under a 12-h light–dark cycle with food and water available *ad libitum*. All experiments were conducted during the light period. Only adult (> 8 weeks old) male mice of normal appearance and weight were used for all behavioral tests, immunohistochemistry, and electrophysiological experiments. Littermate mice were split into random groups before virus injection.

### Viruses

The viruses (in genomic copies per mL) AAV2/9-EF1α-DIO-GCaMP6s (5.64 × 10^12^), AAV2/9-EF1α-DIO-mCherry (9.0 × 10^12^), AAV2/9-hSyn-DIO-hGtACR1-EGFP (5.0 × 10^12^), AAV2/9-EF1α-DIO-EYFP (4.11 × 10^12^), AAV2/9-hSyn-DIO-hM4Di(Gi)-mCherry (3.10 × 10^12^), and AAV2/9-DIO-mGFP-Synaptophysin-mRuby (8.9 × 10^12^) were from Taitool Bioscience, China. In addition, AAV2/9-EF1α-DIO-hChR2-EYFP (5.4 × 10^12^), rAAV2/9-EF1α-DIO-RVG (5.53 × 10^12^), rAAV2/9-CAG-DIO-TVA-EGFP (5.22 × 10^12^), and RV-ENVA-ΔG-dsRed (3.0 × 10^8^) were from BrainVTA Bioscience, China.

### Stereotaxic Viral Injection

Mice were anesthetized with sodium pentobarbital (75 mg/kg, i.p.). Viruses were injected into brain nuclei using a stereotaxic frame (RWD Life Science). Injections were made with a 10-μL syringe connected to a glass micropipette with a 10–50 μm diameter tip. Syringe pumps (78-8130, KD Scientific, USA) were used to inject the virus at a specific speed (30 nL/min) and volume. After each injection, the syringe was left *in situ* for an additional 10 min to allow diffusion of the virus and then withdrawn slowly.

For fiber photometry, a mixture of rAAV2/9-EF1α-DIO-GCaMP6s and AAV2/9-EF1α-DIO-mCherry (2:1,70 nL) was unilaterally injected into the rostral ZI of SST-Cre mice (anteroposterior (AP): − 1.00 mm; mediolateral (ML): 0.6 mm; dorsoventral (DV): − 4.45 mm relative to bregma). After the virus was expressed for 3 weeks, optical fibers (outer diameter: 200 μm, numerical aperture: 0.37, Inper) were implanted above the rostral ZI unilaterally (100 μm above the viral injection coordinates).

For local optogenetic stimulation, AAV2/9-EF1α-DIO-hChR2-EYFP, AAV2/9-EF1α-DIO-EYFP, or AAV2/9-hSyn-DIO-hGtACR1-EGFP (60 nL) was bilaterally injected into the rostral ZI of SST-Cre mice. After the virus was expressed for 3 weeks, optical fibers (outer diameter: 200 μm, numerical aperture: 0.37, Inper) were implanted at a 10° angle relative to the vertical plane above the rostral ZI bilaterally (AP: − 1.00 mm; ML: ± 1.35 mm; DV: − 4.13 mm).

For local pharmacological experiments, AAV2/9-hSyn-DIO-hM4Di-mCherry or AAV2/9-EF1α-DIO-EYFP (60 nL) was bilaterally injected into the rostral ZI of SST-Cre mice. The virus was expressed for 3 weeks before behavioral tests.

For optogenetic stimulation of axon terminals in the nucleus reuniens (Re), AAV2/9-EF1α-DIO-hChR2-EYFP, AAV2/9-EF1α-DIO-EYFP, or AAV2/9-hSyn-DIO-hGtACR1-EGFP (60 nL) was bilaterally injected into the rostral ZI of SST-Cre mice. After the virus was expressed for 3 weeks, optical fibers (outer diameter: 200 μm, numerical aperture: 0.37, Inper) were implanted at a 10° angle relative to the vertical plane above the Re bilaterally (AP: − 0.65 mm; ML: ± 0.93 mm; DV: − 4.0 mm).

For anterograde tracing, AAV2/9-DIO-mGFP-Synaptophysin-mRuby (70 nL) was unilaterally injected into the rostral ZI of SST-Cre mice. Three weeks after the injection, mice were euthanized with sodium pentobarbital (75 mg/kg, i.p.) and then transcardially perfused with phosphate-buffered saline (PBS), followed by 4% paraformaldehyde (PFA) in PBS. Then brain sections were collected for confocal imaging.

For the monosynaptic retrograde tracing experiments, a viral cocktail (1:1, 50 nL) of rAAV2/9-EF1α-DIO-RVG and rAAV2/9-CAG-DIO-TVA-EGFP was unilaterally injected into the rostral ZI of SST-Cre mice for initial infection of starter SST-positive neurons in the rostral ZI. Two weeks later, the same location was injected with the modified rabies virus RV-ENVA-ΔG-dsRed (80 nL). The mice were euthanized 1 week after rabies virus injection and brain sections were cut for confocal imaging.

To trace the monosynaptic upstream nucleus of the rostral ZI-Re pathway during a looming stimulus, a viral cocktail (1:1, 50 nL) of rAAV2/9-EF1α-DIO-RVG and rAAV2/9-CAG-DIO-TVA-EGFP was unilaterally injected into the rostral ZI of SST-Cre mice for initial infection of starter SST-positive neurons in the rostral ZI. Two weeks later, the modified rabies virus RV-ENVA-ΔG-dsRed (80 nL) was injected into the Re (AP: − 0.60 mm; ML: ± 0.2 mm; DV: − 4.40 mm). One week after the injection of modified rabies virus, the mice were exposed to a looming stimulus and brain sections were cut for c-Fos staining.

### *In Vivo* Optogenetic Stimulation

Mice were allowed to recover from optical fiber implantation surgery for 1 week. For optogenetic manipulation during the behavioral assessments, pulses of 473 nm laser stimulation were set by a laser stimulator (Inper) and applied through an implanted fiber (diameter: 200 μm, numerical aperture: 0.37 NA; Inper) to selected brain regions. The laser power was measured at the tip of the fiber and was adjusted to 3–5 mW for optogenetic inhibition (GtACR1-expressing neurons, 2-s pulses at 0.25 Hz or 4 s of continuous light) or 8–10 mW for optogenetic activation (channelrhodopsin-2 (ChR2)-expressing neurons,10-ms pulses at 20 Hz).

### Dual Color Fiber Photometry

The dual color fiber photometry system (ThinkerTech Nanjing Bioscience) had two excitation light sources, 480 nm and 570 nm, the former was used to record GCaMP6s fluorescent signals and the latter was used to record mCherry fluorescent signals as reference to effectively discriminate motion artifacts and real Ca^2+^ fluorescent signals. Averaged traces of Ca^2+^ fluorescent signal changes and heatmaps were plotted using the MatLab program provided by ThinkerTech Nanjing Bioscience.

### Behavioral Paradigms

One week after implantation of the optical fibers, the mice were handled daily for 3 days. Before behavioral tests, the mice were habituated in the test room for at least 30 min before each behavioral experiment started to minimize the effects of stress. The apparatus was cleaned with 75% ethanol to eliminate odor from other mice. Experimenters were blinded to all training and behavioral assessments.

### Open Field Test

Mice were placed in the center zone of an open field arena (45 cm × 45 cm) and allowed to move freely for a 12-min session which divided into four 3-min epochs. The epochs alternated between light stimulation OFF and light stimulation ON periods, beginning with a light OFF epoch. The paths of the mice were tracked by ANY-Maze software.

### Elevated Plus Maze Test

Mice were placed in the center zone of an elevated plus maze (EPM) with the head towards the open arm area and allowed to move freely for a 12-min session divided into four 3-min epochs. The epochs alternated between light stimulation OFF and light stimulation ON periods, beginning with a light OFF epoch. The paths of the mice were tracked by ANY-Maze software.

### Rotarod Test

Mice were trained for 3 consecutive days from 5 to 13 r/min. Mice that were able to stay on the rotating rod at 13 r/min for 60 s were used in the behavioral tests. In the behavioral tests, mice from same cage were placed in separate lanes on a rotating rod that was set to accelerate from 4 to 40 r/min in 300 s. The test procedure was repeated for 6 trials separated by 15-min inter-trial intervals. In the optogenetic stimulation test, 6 epochs alternated between light OFF and light ON periods, beginning with a light OFF epoch. Blue light was applied continuously until the mice fell from the rotating rod during light ON periods.

### Looming Test

The looming test was applied in a custom-built 35 cm × 35 cm × 30 cm closed box with a shelter nest in the corner and an LCD monitor placed on the top of the box to display the looming stimulus. Each mouse was habituated to the looming box for 10 min 1 day before testing and the mice were acclimated to the arena for 5–8 min before stimulus display. Then, the looming stimulus was initiated when the mouse was in the center of the arena. The looming visual stimulus was applied through a computer monitor directly above the animal as an expanding black disc from a diameter of 2° to 20° of visual angle within 250 ms, and remained at 20° visual angle for 250 ms. This stimulus was repeated 3 times at 0.5-s intervals (3 s in total).

Before optogenetic and pharmacological stimulation in the looming test, we applied the looming stimulus pre-test 1 week before formal experiments to ensure the defensive behavior was induced by the stimulus. Mice that showed no defensive responses to the looming stimulus in the pre-test were excluded from subsequent experiments. In the optogenetic, c-Fos, and pharmacological stimulation experiments plus the looming experiment, we applied 5 trials of the looming stimulus while only the first two defensive behavioral outputs were analyzed; in the fiber photometry experiment, 3 trials of the looming stimulus were presented and analyzed. No adaptation was found in any of our experiments. In the optogenetic inhibition experiments, light stimulation was delivered 1 s before the onset of the looming stimulus and continued until the stimulus was turned off. In the optogenetic activation experiments, light stimulation was delivered 1 s before the onset of the looming stimulus and lasted for 8 s in one trial of the behavioral test. And in the pharmacological experiments, clozapine *N*-oxide (CNO, Sigma, 5 mg/kg, i.p.) was administered 30 min before the looming test. Behavior was recorded with two HD digital cameras (C920, Logitech) and data were analyzed with Anymaze software.

### Sweeping Test

Mice were placed in the same chamber as that used for the looming behavior and were habituated to the arena for 10 min before the behavioral test. Then, the sweeping stimulus was applied as a 2.5-cm diameter black disc moving across the screen when the mouse was in the center of the arena. All mice were tested only once owing to the fact that they habituated to this stimulus even after one trial. The sweeping stimulus lasted for 4 s in the fiber photometry experiment, and for 6 s in the optogenetic inhibition experiment [27]. Behavior was recorded with two HD digital cameras (C920, Logitech) and data were analyzed with Anymaze software.

### Electrocardiogram Recording

Heart rate recordings in conscious animals were measured using the MouseOXPlus non-invasive pulse oximeter (Starr Life Sciences, Oakmont, PA). The neck collar and system were set up according to the manufacturer instructions. Each mouse was acclimated to the neck collar for 10 min 1 day before testing. In optogenetic experiments, the heart rate was recorded for a 1-min light OFF epoch followed by a 1-min ON epoch and repeated for 3 trails.

### Histological Analysis and Imaging

Mice were anesthetized with sodium pentobarbital (75 mg/kg, i.p.) and then transcardially perfused with PBS, followed by 4% PFA in PBS. The brain was then removed and post-fixed in 4% PFA for 6–8 h at 4 °C, and dehydrated in 30% sucrose (wt/vol) in PBS for 48 h. The brains were embedded in Optimal Cutting Temperature compound and coronal cryosections were cut at 50 μm on a cryostat (CM1950, Leica Microsystems, Germany).

The brain sections were washed three times with PBST (0.3% Triton X-100 in PBS) for 5 min each, then blocked with 5% bovine serum albumin in PBST for 1 h and incubated with primary antibodies overnight at 4 °C. After incubation, the sections were washed and incubated with a fluorescent dye-conjugated secondary antibody (1:400, Invitrogen, USA) for 1 h at room temperature. The primary antibodies were anti-glutamate (1:1000, G6642, Sigma-Aldrich, USA), anti-GABA (1:1000, G6642, Sigma-Aldrich), and anti c-Fos (1:1000, 226003, Synaptic Systems, Germany, or 1:500, 226004, Jackson ImmunoResearch, USA). Sections were mounted after staining with the nuclear dye 4,6-diamidino-2-phenylindole (DAPI, 1:5000 of 5 mg/mL, Sigma-Aldrich).

After mounting, the sections were scanned and imaged under a 10× objective using a Virtual Slide Microscope VS120 (Olympus). Confocal images were captured under a 10× or 20× objective (numerical aperture 1.2) using an A1R Confocal Microscope (Nikon, Japan) and were processed using ImageJ. To quantify the number of neurons in different brain regions that projected to the SST-positive neurons in the rostral ZI, we counted the RV-labeled cells and manually registered the location to the mouse brain atlas in every other section along the rostral–caudal axis.

### RNAscope *In Situ* Hybridization

Coronal sections (16 μm) were prepared as described above. Fluorescence *in situ* hybridization was applied according to the manufacturer’s standard protocols with the RNAscope multiplex fluorescent reagent kit v2 (RNAscope Probe-Mm-Sst, Cat No. 404631) and probes from ACDbio (Minneapolis, USA).

### Electrophysiology

Mice with AAV-virus-mediated ChR2 or GtACR1 expression in SST-positive neurons in the rostral ZI were anesthetized with pentobarbital sodium (75 mg/kg, i.p.) followed by transcardial perfusion with ~ 20 mL of ice-cold oxygenated (95% O_2_ and 5% CO_2_) *N*-methyl-D-glucamine (NMDG)-HEPES artificial cerebrospinal fluid (aCSF) composed of the following (in mmol/L): 92 NMDG, 2.5 KCl, 1.25 NaH_2_PO_4_, 30 NaHCO_3_, 20 HEPES, 25 glucose, 2 thiourea, 5 Na-ascorbate, 3 Na-pyruvate, 0.5 CaCl_2_·2H_2_O, and 10 MgSO_4_·7H_2_O. Coronal brain slices with the rostral ZI and Re were cut at 250 µm on a vibratome (VT1200s, Leica, Germany) in ice-cold oxygenated NMDG-HEPES aCSF and then incubated in NMDG-HEPES oxygenated aCSF at 34 °C for 30 min. The incubation aCSF was spiked with 250, 250, 500, 1000 and 2000 µL of NaCl solution (0.78 g NaCl dissolved in 5 mL freshly-prepared NMDG-HEPES aCSF, Na^+^ spike-in solution) at 5, 10, 15, 20, and 25 min, respectively [28]. Then the slices were transferred to oxygenated HEPES holding aCSF (in mmol/L, 92 NaCl, 2.5 KCl, 1.25 NaH_2_PO_4_, 30 NaHCO_3_, 20 HEPES, 25 glucose, 2 thiourea, 5 Na-ascorbate, 3 Na-pyruvate, 2 CaCl_2_·2H_2_O, and 2 MgSO_4_·7H_2_O) and incubated for at least 1 h at room temperature before recording.

Slices were then transferred to a recording chamber with continuously-oxygenated recording aCSF containing (in mmol/L): 124 NaCl, 2.5 KCl, 1.25 NaH_2_PO_4_, 24 NaHCO_3_, 12.5 glucose, 5 HEPES, 2 CaCl_2_·2H_2_O, and 2 MgSO_4_·7H_2_O. The signals were amplified by a MultiClamp 700B amplifier, sampled at 10 kHz with a Digidata 1440A analog-to-digital converter, and filtered at 2 kHz. Fluorescently-labeled cells were visualized under a 40× water-immersion objective (Nikon Eclipse FN1 microscope, Japan). Data were collected 2 min after obtaining stable whole-cell parameters with pClamp 10.4 software (Molecular Devices, USA). If series resistance changed by > 20% during recording, the data were abandoned. To test the monosynaptic and inhibitory connection of the rostral ZI-Re, light-evoked inhibitory postsynaptic currents (eIPSCs) were recorded at − 70 mV using electrodes filled with high chloride internal solution contained (in mmol/L): 110 potassium gluconate, 40 KCl, 10 HEPES, 3 Mg-ATP, 0.5 Na_3_-GTP, and 0.2 EGTA. Tetrodotoxin (TTX, 1 μmol/L, Tocris, UK), and 4-aminopyridine (4-AP, 100 μmol/L, Sigma-Aldrich, USA) were used to block multi-synaptic connections. picrotoxin (PTX, 50 μmol/L, Tocris, UK) was added to block the GABA_A_R-mediated eIPSCs. To test the virus efficiency of AAV-DIO-ChR2, current-clamp mode was used to record the action potentials of ChR2-expressing neurons induced by blue light (473 nm, 5-ms pulses, ~ 10 mW) at 20 Hz. The baseline membrane potential was held at − 45 mV. The low-chloride internal solution contained (in mmol/L): 150 potassium gluconate, 5 NaCl, 10 HEPES, 1 MgCl_2_, 2 Mg-ATP, 0.5 Na_3_-GTP, and 0.2 EGTA (pH 7.3). For AAV-DIO-GtACR1, the GtACR1-expressing neurons were recorded for 3 s as baseline, followed by 5 s of continuous blue light stimulation (473 nm, ~ 5 mW) and 5 s of recovery. Data were measured with Clampfit v10.4.

### Statistical Analysis

MatLab 2014a and GraphPad Prism 6 were applied for statistical analysis. The following statistical tests were used for the behavioral data analyses: two-tailed *t*-test, one-way analysis of variance (ANOVA), or two-way ANOVA. The *post hoc* analysis was applied using Sidak's multiple comparisons test when indicated. Data are presented as the mean ± SEM.

## Results

### SST-Positive Neurons in the Rostral ZI are Activated by Looming Stimulus-Evoked Defensive Behaviors

**Fig. 1:**
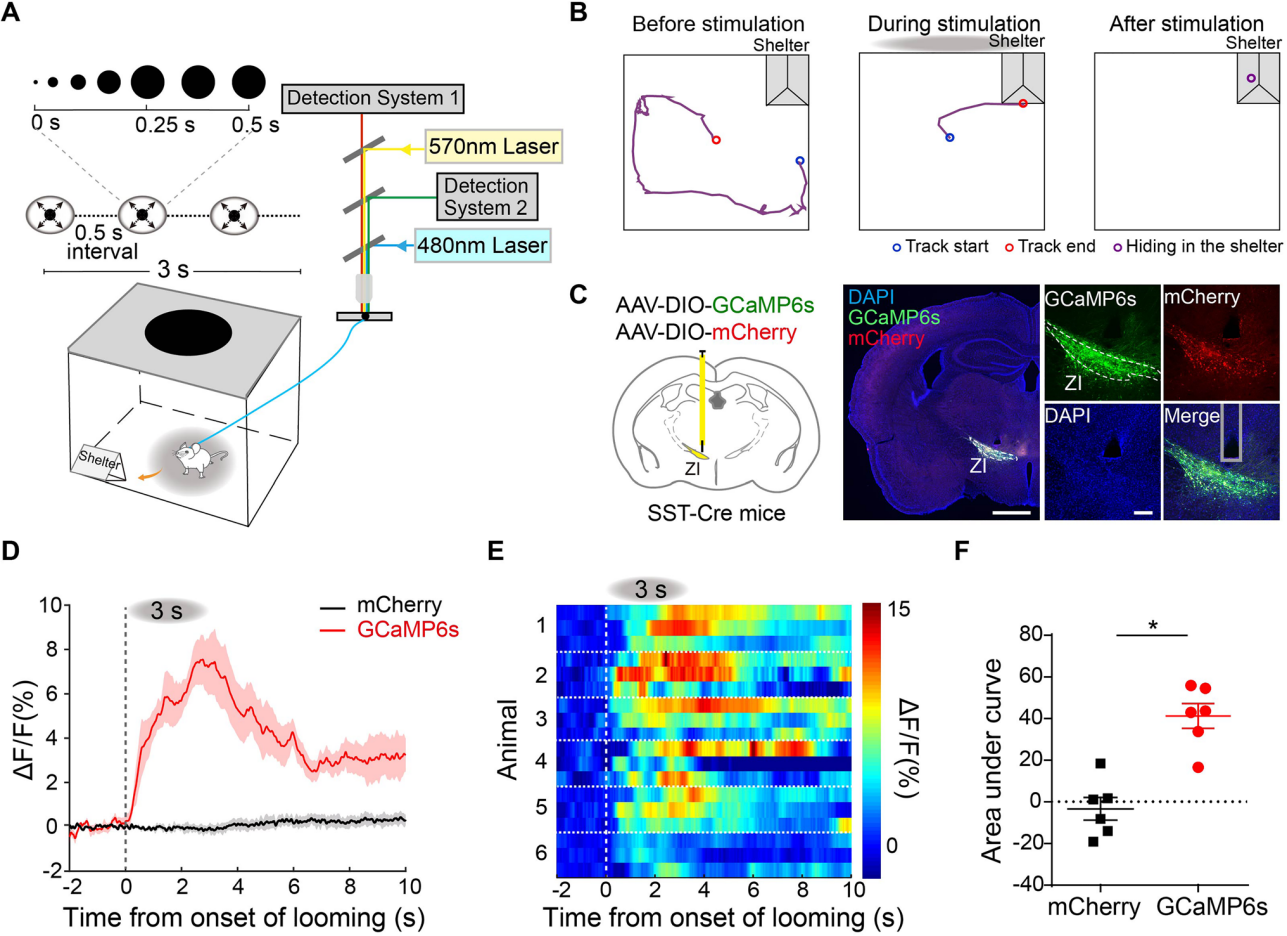
SST-positive neurons in the rostral ZI are activated in looming stimulus-evoked defensive behaviors. **A** Schematics of overhead looming stimulus-evoked defensive behavior in a shelter-containing open field apparatus. *In vivo* Ca^2+^ signals recorded by dual-color fiber photometry. **B** Representative traces of movement before (30 s, left), during (3 s, middle), and immediately after a looming stimulus (10 s, right). **C** Left: AAV-DIO-GCaMP6s and AAV-DIO-mCherry injection into the rostral ZI of SST-Cre mice. Right: representative images of GCaMP6s and mCherry expression in the rostral ZI of SST-positive neurons. Gray column: position of the fiber track. Scale bars, 1 mm (left) and 200 μm (right). **D** Average change of Ca^2+^ fluorescence intensity of freely-moving mice during a looming stimulus. Gray dashed line: onset of looming stimulus; red, GCaMP6s virus channel recording; black, mCherry control virus channel recording; shaded areas, standard error of the mean (SEM). **E** Representative trial-by-trial heat map of Ca^2+^ transients evoked by a looming stimulus in 18 trials from 6 mice (3 trials per mouse). Vertical white dashed line, onset of the looming stimulus. **F** Statistical analysis of area under the curve (AUC) of average Ca^2+^ transients induced by a looming stimulus in the GCaMP6s and mCherry groups (*n* = 6 mice, *P = 0.0313, two-tailed paired *t* test). Data are presented as the mean ± SEM.

Innate defensive behaviors can be triggered by either alarming visual cues or an olfactory threat emitted by predators. To investigate whether SST-positive neurons in the rostral ZI participated in innate visual threat-evoked defensive behaviors, we set up an overhead looming stimulus paradigm [18, 21] to mimic approaching aerial predators in the wild. Mice were placed in an open field with a shelter in the corner and an overhead expanding dark disc (looming stimulus) projected by a monitor. The looming stimulus including one cycle of a dark disk expanding from 2° to 20° visual angle in 250 ms and remained at 20° for additional 250 ms, repeated 3 times at 500-ms intervals (Fig. 1A). We found that mice exhibited reliable flight behavior upon the looming stimulus, which triggered immediate flight to and hiding in the shelter (Fig. 1B; video 1).

To monitor the activity of SST-positive neurons in the rostral ZI during a looming stimulus, we selectively expressed genetically-encoded Ca^2+^ indicators (GCaMP6s) and mCherry as control in the rostral ZI by Cre-dependent adeno-associated viruses (AAV) in SST-Cre mice (Fig. 1C). We first assessed the abundance of SST mRNA in brain slices from SST × Ai14 mice with *in situ* hybridization to confirm the SST-Cre line, and found that SST mRNA was strongly expressed in SST-positive neurons in the rostral ZI (Fig. S1). We used dual-color fiber photometry to record the population activity changes of Ca^2+^ signals in these neurons (Figs. 1A and S10A). At the onset of the looming stimulus, we found an increase in the intensity of GCaMP6s fluorescence in SST-positive neurons in the rostral ZI but found no changes in fluorescent intensity in the control mCherry channel (Fig. 1D–F).

To investigate whether SST-positive neurons in the rostral ZI can be activated by other exteroceptive stimuli that induced innate defensive behavior, we examined the GCaMP6s fluorescence change in these neurons in response to an exteroceptive stimulus. We found that a sweeping stimulus [27], which induces freezing but not flight behavior in mice, also induced intracellular Ca^2+^ elevation in SST-positive neurons in the rostral ZI (Fig. S2A). By contrast, we did not find an increase of the Ca^2+^ signal during normal locomotion in free-moving mice (Fig. S2B). And we also recorded the Ca^2+^ signals of other neuronal subtypes in the rostral ZI in response to a looming stimulus. We found that exposure to looming stimulus did not increase the activity of PV-positive neurons in the rostral ZI (Fig. S2C). Together, these results demonstrated that SST-positive neurons in the rostral ZI are activated in visual cue-induced innate defensive behaviors.

### Inhibition of SST-Positive Neurons in the Rostral ZI Attenuates Flight Response to a Looming Stimulus

**Fig. 2:**
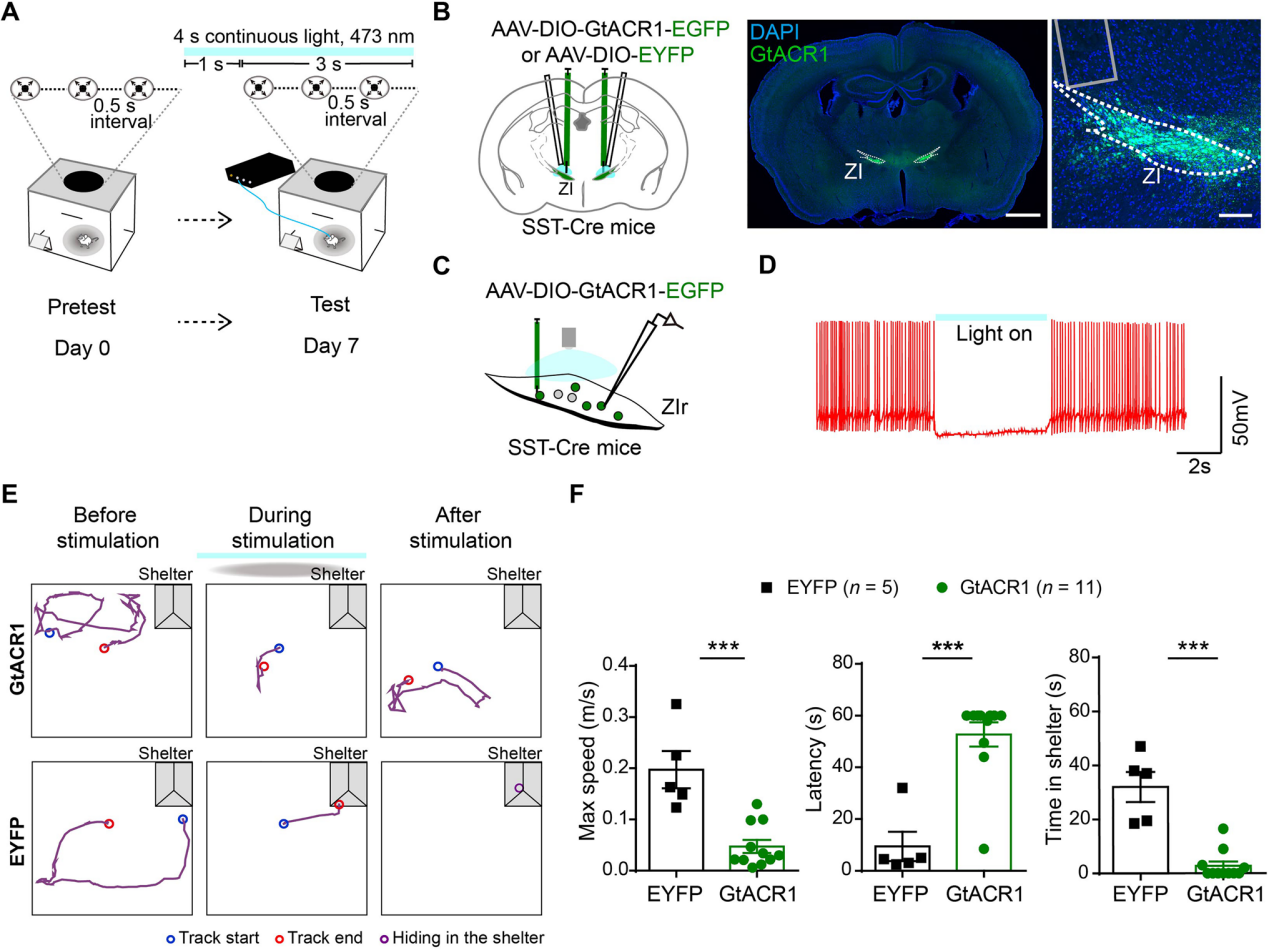
Optogenetic inhibition of SST-positive neurons in the rostral ZI attenuates looming stimulus-evoked defensive behaviors. **A** Schematic of optogenetic manipulation of bilateral rostral ZI SST-positive neurons during the looming stimulus on pre-test and test days. Continuous blue light stimulation is delivered through optic fibers 1 s before the onset of the looming stimulus and lasts for 4 s on test day. **B** Schematic (left) and representative images (right) showing bilateral optogenetic stimulation and labeling with Cre-dependent AAV-mediated GtACR1-EGFP or EYFP expression in the rostral ZI in SST-Cre mice. Gray column: position of the fiber track. Scale bars, 1 mm (left) and 100 μm (right). **C** Schematic and representative whole-cell recording trace (**D**) showing that light stimulation (5 s continuous light, 473 nm laser) inhibits the action potential firing of GtACR1-expressing SST-positive neurons in the rostral ZI. **E** Representative traces of movement of the GtACR1 group (upper row) and EYFP group (lower row) before (30 s, left), during (3 s, middle) and immediately after a looming stimulus (10 s, right). **F** Statistical analysis of the maximum speed, latency to the shelter, and hiding time spent in the shelter upon a looming stimulus and optogenetic stimulation in the GtACR1-group compared with EYFP group. Max speed, ***P = 0.0007; latency, ***P = 0.0005; time in shelter, ***P = 0.0002; two-tailed unpaired *t* test.

To explore whether endogenous activity of SST-positive neurons in the rostral ZI can modulate looming stimulus-evoked defensive behaviors, we optogenetically suppressed these neurons by bilaterally injection of AAV virus carrying Cre-dependent *Guillardia theta* anion channel rhodopsin 1 protein (GtACR1) [29] or enhanced yellow fluorescent protein (EYFP) into the rostral ZI of SST-Cre mice (Fig. 2A–C and S10C). We tested the efficiency of GtACR1-mediated inhibition in acute brain slices using whole-cell recordings and found that continuous blue light (473 nm) stimulation induced membrane hyperpolarization and decreased the neuronal action potential firing in SST-positive neurons in the rostral ZI (Fig. 2D). At least a week before subsequent behavioral tests, all mice were exposed to the looming stimulus to examine whether defensive behavior could be induced (pre-test). Mice that showed no defensive responses upon looming stimulus in the pre-test were excluded from subsequent experiments. In addition, there was no difference in the maximum speed of mice in flight response upon looming stimulus during the pre-test when comparing with the maximum speed of flight response during formal test (Fig. S3).

Next, we examined the effect of optogenetic suppression of SST-positive neurons in the rostral ZI during the looming stimulus (Fig. 2A). We found that inhibition of these neurons resulted in decreased maximum speed of flight, increased latency for returning to the shelter, and reduced time spent hiding in the shelter after the looming stimulus in GtACR1 group comparing with the EYFP group (Fig. 2E, F; videos 2 and 3). In addition, we used pharmacological inhibition to suppress SST-positive neurons in the rostral ZI by bilaterally injection of AAV virus carrying the Cre-dependent hM4Di (G_i/o_-coupled human muscarinic M4 designer receptor exclusively activated by a designer drug, DREADD) or control EYFP into the rostral ZI of SST-Cre mice (Fig. S4A, B). The pharmacological hM4Di receptor ligand clozapine-N-oxide (CNO) was administered 30 min before the looming stimulus test by intraperitoneal injection. Behavioral results showed that mice still showed flight response upon the looming stimulus after pharmacological inhibition of SST-positive neurons in the rostral ZI. There was no significant difference in the maximum speed of flight or latency for returning to the shelter, but reduced time spent in the shelter after the looming stimulus was observed in hM4Di group comparing with EYFP group (Fig. S4D, E). Thus, pharmacological inhibition of SST-positive neurons in the rostral ZI (Fig. S4D, E) showed less significant effect in blocking flight responses, possibly due to the relatively low efficiency of the suppressing action potential firing of SST-positive neurons by hM4Di-mediated inhibition in comparison with optogenetic silencing (Figs. 2D and S4C). Together, these data suggest that the activity of SST-positive neurons in the rostral ZI is required for looming stimulus-induced innate defensive behaviors.

### Activation of SST-Positive Neurons in the Rostral ZI Induces Freezing-Like Defensive Behaviors

**Fig. 3:**
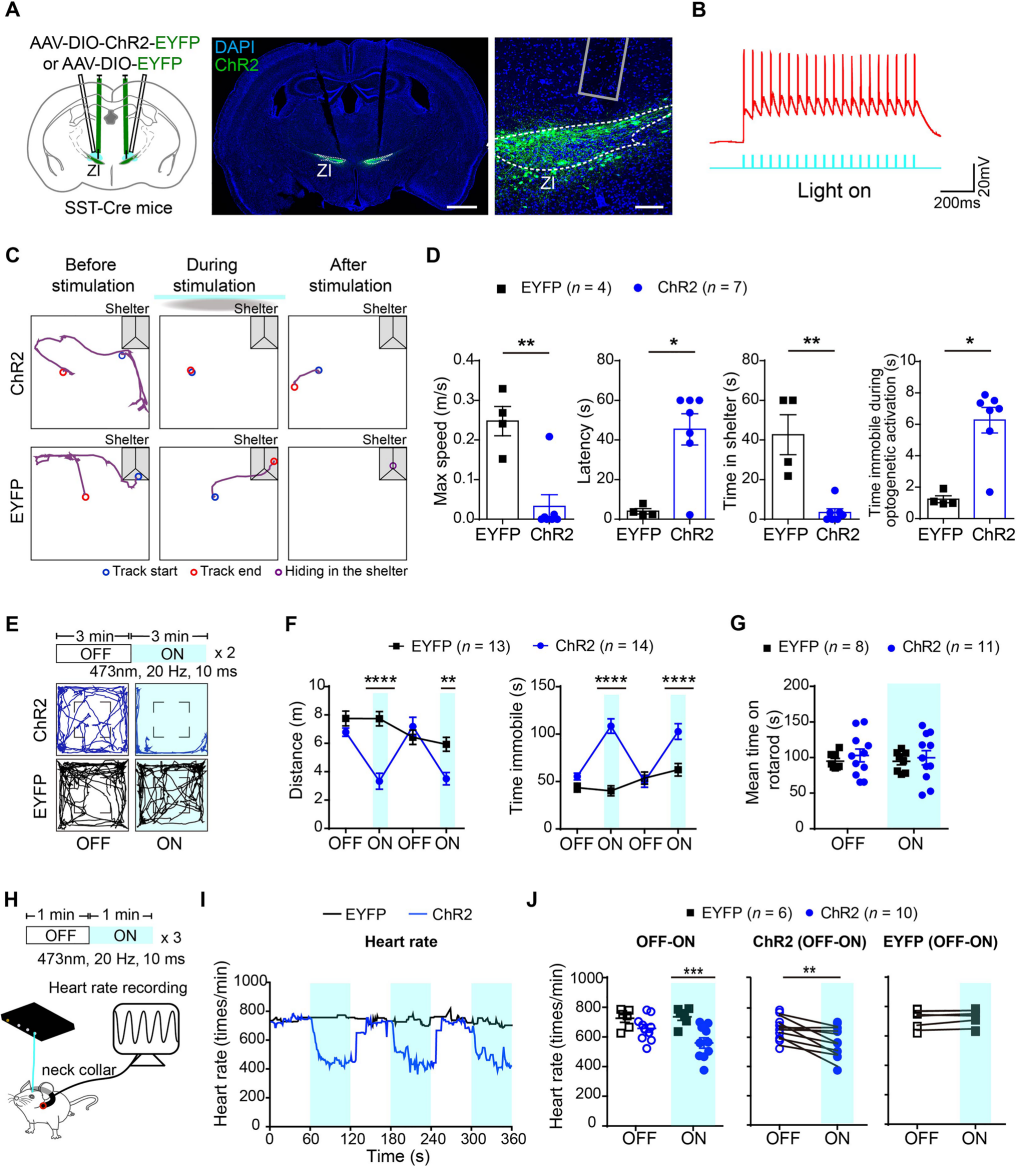
Optogenetic activation of SST-positive neurons in the rostral ZI induces freezing-like defensive behavior. **A** Schematic (left) and representative images (right) showing bilateral optogenetic stimulation and labeling with Cre-dependent AAV-mediated ChR2-EYFP or EYFP expression in the rostral ZI in SST-Cre mice. Gray column: position of the fiber track. Scale bars, 1 mm (left) and 100 μm (right), respectively. **B** Representative trace of action potential firing evoked by 20-Hz optogenetic stimulation in ChR2-expressing SST-positive neurons in the rostral ZI. **C** Representative traces of movement in the ChR2 group (upper) and EYFP group (lower) before (30 s, left), during (3 s, middle) and immediately after a looming stimulus (10 s, right). **D** Statistical analysis of the maximum speed, latency to the shelter, and hiding time spent in the shelter upon the looming stimulus and optogenetic stimulation, and immobile time during optogenetic stimulation in the ChR2 group compared with the EYFP group. Maximum speed, **P = 0.0091; latency, *P = 0.0424; time in shelter, **P = 0.0061; time immobile during optogenetic activation, *P = 0.0121, two-tailed unpaired *t* test. **E** Representative traces and statistical analysis (**F**) showing the movement of mice in the open field test upon light stimulation of the ChR2 group (upper) and EYFP group (lower). Locomotor distance (left in **F**), *F*_(3,75)_ = 11.89, *P* < 0.0001. ****P < 0.0001, **P = 0.0038 for laser ON stage comparison; immobile time (right in **F**), *F*_(3,75)_ = 14.58, *P* < 0.0001. ****P < 0.0001. **G** Statistical analysis of rotarod test upon light stimulation of ChR2-expressing neurons in SST-positive neurons in the rostral ZI (*F*_(1,17)_ = 0.07943, *P* = 0.7815, *P* = 0.8962 for laser ON stage comparison). **H** Schematic showing measurement of heart rate during optogenetic stimulation. **I** Representative traces and statistical analysis (**J**) of changes in heart rate upon optogenetic stimulation. OFF–ON: changes in heart rate upon optogenetic stimulation of SST-positive neurons in the rostral ZI in ChR2 group comparing with EYFP group (*F*_(1,14)_ = 11.08, *P* = 0.0050, ***P = 0.0001). ChR2 (OFF–ON): changes in heart rate before and after optogenetic stimulation in the ChR2 group, **P = 0.0032, two-tailed paired *t* test. EYFP (OFF–ON): changes in heart rate before and after optogenetic stimulation in the EYFP group, *P* = 0.1563, two-tailed paired *t* test. Two-way repeated-measures ANOVA and Sidak's multiple comparisons test for panels (**F**, **G**), and left in (**J**). Data are presented as the mean ± SEM.

We next examined whether activation of SST-positive neurons in the rostral ZI can modulate innate defensive behavior in the looming stimulus test. We bilaterally injected Cre-dependent AAV virus carrying ChR2 or control EYFP into the rostral ZI of SST-Cre mice (Figs. 3A and S10B). We tested the efficiency of ChR2 stimulation in acute brain slices and found that blue light stimulation (473 nm, 20 Hz) of ChR2-expressing SST-positive neurons in the rostral ZI evoked reliable action potential firing (Fig. 3B). Next, we assessed the behavior response to looming stimulus when optogenetically activating SST-positive neurons in the rostral ZI *in vivo.* We applied blue light stimulation 1 s before applying the looming stimulus and lasted for 8 s in one trial of the looming stimulus behavioral test. We found that blue light stimulation significantly reduced the locomotor activity of mice that stayed still in the center of the arena despite the looming stimulus (Fig. 3C; videos 4 and 5). We quantified the locomotor activity and found that the optogenetic activation of SST-positive neurons in the rostral ZI resulted in decreased maximum speed of flight, increased latency for returning to the shelter, decreased time spent in the shelter after the looming stimulus, and increased immobile time during optogenetic activation in the ChR2 group *versus* the EYFP group (Fig. 3D).

To explore whether activation of SST-positive neurons might lead to freezing-like behavior, we assessed the behavioral response upon optogenetic activation of SST-positive neurons in the rostral ZI in open field test without any exogenous stimulation. We found that the mice exhibited immobile behavior following optogenetic activation of SST-positive neurons in the rostral ZI in the open field test (Fig. 3E; videos 6 and 7). Decreased locomotor distance and increased immobile time were found in the ChR2 group comparing with the EYFP group during blue light stimulation (Fig. 3F). However, optogenetic activation of SST-positive neurons in the rostral ZI did not change the motor coordination and motor function of ChR2 group *versus* the EYFP group in the rotarod test (Fig. 3G). Fear induces strong bodily responses including body movement and cardiovascular changes [30]. To determine whether optogenetic activation of SST-positive neurons in the rostral ZI induces freezing-like defensive behavior, we measured the heart rate in free-moving mice with a non-invasive pulse oximeter. The mice showed bradycardia as reflected by a gradual but lasting reduced heart rate during light stimulation in the ChR2 group comparing with EYFP group (Fig. 3H–J). To determine whether SST-positive neurons in the rostral ZI is required for the freezing response, we optogenetically inhibited these neurons during a sweeping stimulus (Fig. S5A). We found that inhibition of SST-positive neurons in the rostral ZI resulted in decreased freezing time after the sweeping stimulus in GtACR1 group in comparison with the EYFP group (Fig. S5B, C). Together, these finding indicated that optogenetic activation of SST-positive neurons in the rostral ZI induces immobility and bradycardia that resembles the freezing-like defensive behavior when mice encounter an approaching predator.

### SST-Positive Neurons in the Rostral ZI-Re Pathway Mediate Looming Stimulus-Evoked Defensive Behaviors

**Fig. 4:**
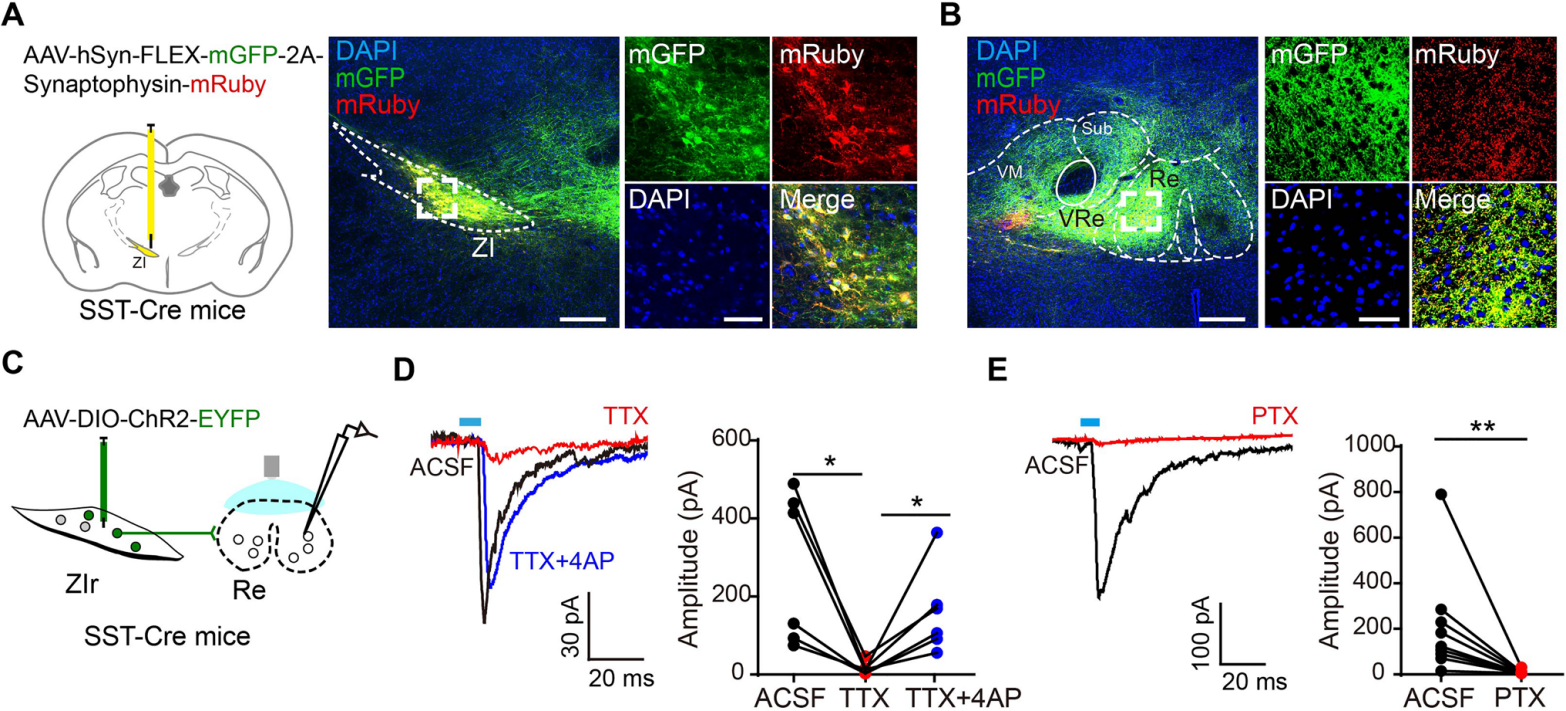
Direct monosynaptic GABAergic projection from SST-positive neurons in the rostral ZI to nucleus reuniens. **A** Schematic (left) and representative images (right) showing unilateral Cre-dependent AAV mediated mGFP and synaptophysin-mRuby expression in the rostral ZI in SST-Cre mice. Scale bars, 200 μm (left) and 50 μm (right). **B** Representative images showing the axon terminal expression of mGFP and mRuby in the Re from SST-positive neurons in the rostral ZI. Scale bars, 200 μm (left) and 50 μm (right). **C** Schematic of electrophysiological recording synaptic currents of Re neurons upon optogenetic stimulation of axon terminals from SST-positive Re-projection neurons in the rostral ZI. **D** Representative traces (left) and statistical analysis (right) of optogenetic stimulation evoked IPSCs in Re neurons in ACSF and following sequential application of TTX (1 μmol/L) or TTX + 4-AP (100 μmol/L), respectively. ACSF *versus* TTX, *P = 0.0313, TTX *v.* TTX + 4AP, *P = 0.0313, two-tailed paired *t* test. *n* = 6 neurons from 3 mice. **E** Representative traces (left) and statistical analysis (right) of optogenetic stimulation evoked IPSCs recorded in Re neurons in ACSF and following application of PTX (50 μmol/L), respectively. Blue bar indicated optogenetic light stimulation. (**P = 0.0039, *n* = 9 neurons from 3 mice, two-tailed paired *t* test).

We expressed membrane-bound GFP (mGFP) and synaptophysin-mRuby (SYP-mRuby) viruses [31] in SST-positive neurons in the rostral ZI to investigate the possible downstream projections that mediate the looming stimulus-induced defensive behaviors (Fig. 4A). We examined the axon terminal projections in the whole brain and found dense axon terminals in midline thalamic nuclei and the Re (Fig. 4B). In the rodent brain, the Re consists of glutamatergic neurons and has been implicated in several functions ranging from spatial working memory processing [32, 33], stress and depression [34], and the modulation of visually-induced defensive behaviors [23, 25]. Thus, we investigated whether the projection from SST-positive neurons in the rostral ZI to the Re (ZIr^SST^-Re pathway) mediates defensive behaviors. We found the mGFP and synaptophysin-mRuby expressing axon terminals projected to the glutamatergic neurons in the Re (Fig. S6). To further confirm that Re received functional GABAergic projections from SST-positive neurons in the rostral ZI, we recorded blue light-evoked IPSCs (eIPSC) in neurons in the Re when optogenetically activating the axonal terminals in the rostral ZI-Re pathway in brain slices. The eIPSCs were completely blocked by the voltage-gated Na^+^ channel blocker TTX, but were rescued by application of the voltage-gated K^+^ channel blocker 4-AP (Fig. 4C, D). In addition, the eIPSCs were fully abolished by the GABA_A_ receptor antagonist PTX (Fig. 4E). These results demonstrated direct monosynaptic GABAergic projections from SST-positive neurons in the rostral ZI to the Re.

**Fig. 5:**
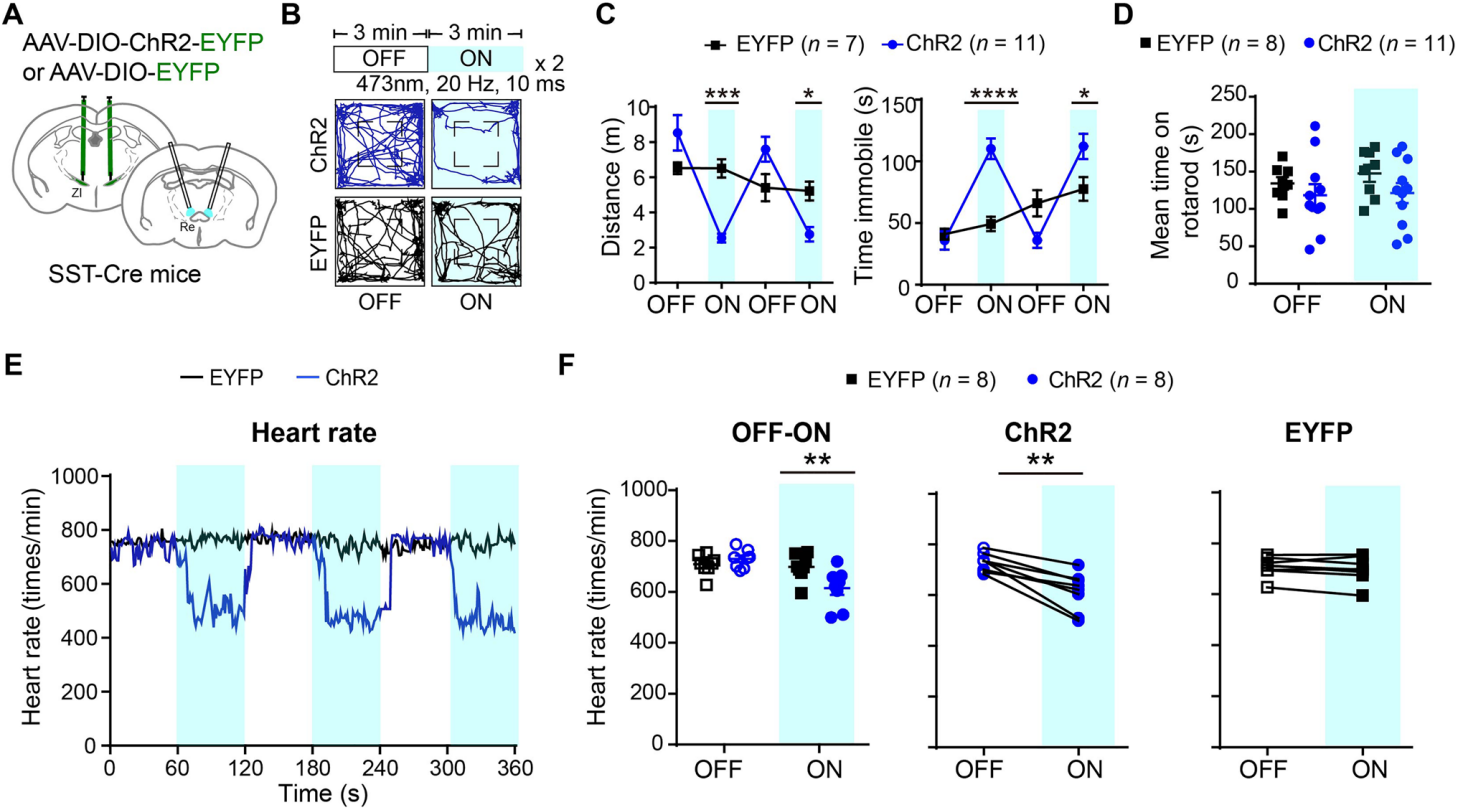
Activation of axon projections from SST-positive neurons in the rostral ZI to Re induces freezing-like behavior. **A** Schematic showing bilateral optogenetic stimulation of axon terminals in the Re with AAV mediated Cre-dependent expression of ChR2 or EYFP in the rostral ZI in SST-Cre mice. **B** Representative traces and statistical analysis (**C**) of optogenetic activation of SST-positive neurons in the rostral ZI-Re pathway in the open field test in the ChR2 (upper) and EYFP (lower) groups. Locomotor distance, *F*_(3,48)_ = 16.71, *P* < 0.0001. ***P = 0.0004, *P = 0.0460; immobile time, *F*_(3,48)_ = 14.19, *P* < 0.0001. ****P < 0.0001, *P = 0.0229. **D** Statistical analysis of the performance in the rotarod test upon optogenetic activation of SST-positive neurons in the rostral ZI–Re pathway (*F*_(1,17)_ = 0.3350, *P* = 0.5703, *P* = 0.3086 for laser ON stage comparison). **E** Representative traces and statistical analysis (**F**) of changes in heart rate upon optogenetic activation of SST-positive neurons in the rostral ZI-Re pathway in ChR2 comparing with EYFP group (left, *F*_(1,14)_ = 20.54, *P* = 0.0005, **P = 0.0075), as well as before or after optogenetic stimulation in the ChR2 group (middle, **P = 0.0012) and EYFP group (right, *P* = 0.1688), two-tailed paired *t* test. Two-way repeated-measures ANOVA and Sidak's multiple comparisons test for panels **C**, **D**, and left in **F**. Data are presented as the mean ± SEM.

**Fig. 6:**
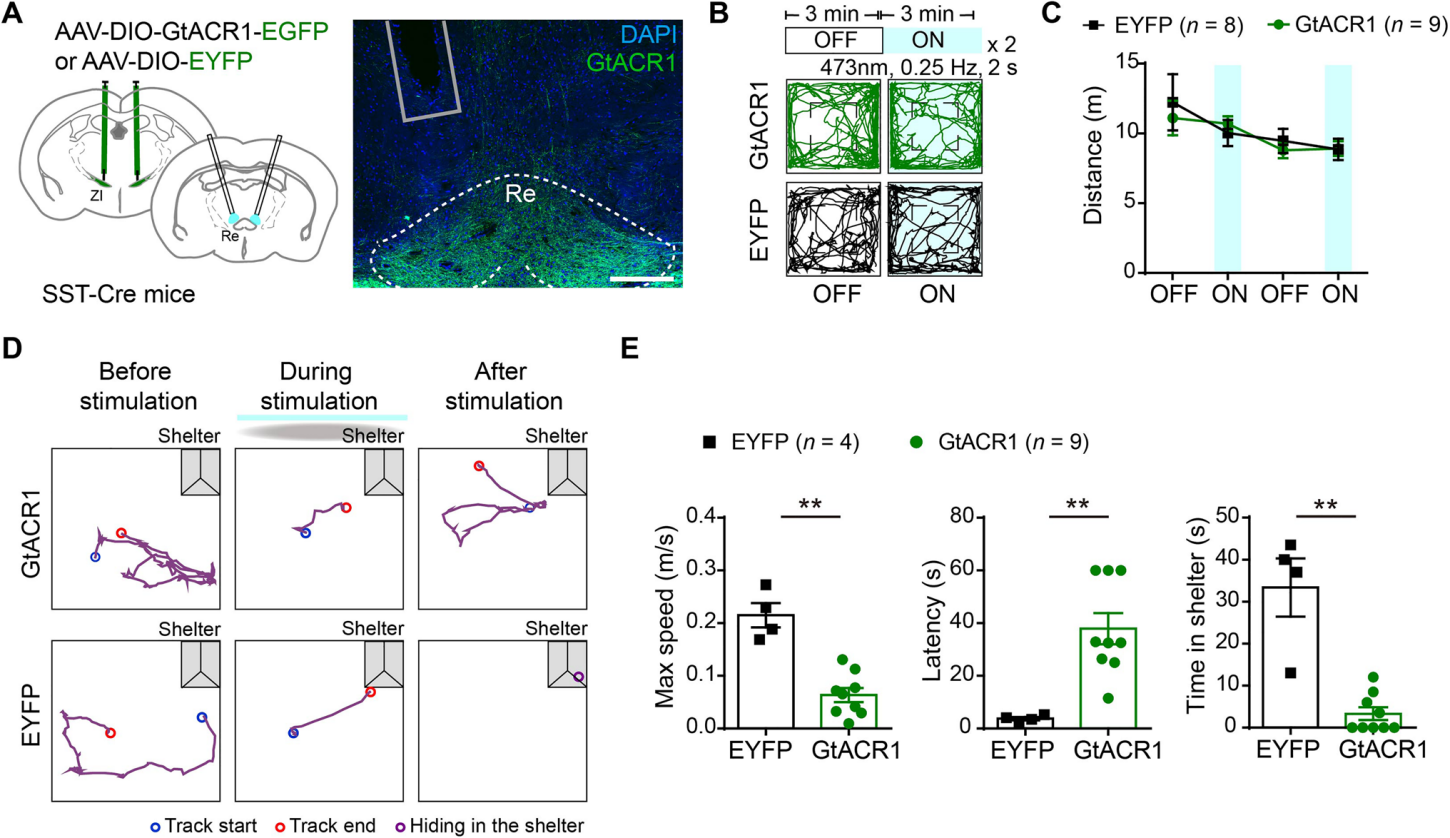
Optogenetic inhibition of axon projections from SST-positive neurons in the rostral ZI to the Re attenuates looming stimulus-evoked defensive behaviors. **A** Schematic showing bilateral Cre-dependent AAV-mediated GtACR1 or EYFP expression in the rostral ZI and optogenetic stimulation of the axon terminals in the Re. Scale bar, 200 μm. **B** Representative traces and statistical analysis (**C**) of locomotor distance in the open field test upon optogenetic inhibition of SST-positive neurons in the rostral ZI–Re pathway in the GtACR1 (green) and EYFP (black) groups, respectively. Locomotor distance, *F*_(3,54)_ = 0.6720, *P* = 0.5729. **D** Representative traces and statistical analysis (**E**) of movement upon looming stimulus when optogenetically stimulating SST-positive neurons in the rostral ZI–Re pathway in the GtACR1 and EYFP groups, respectively. Representative traces of movement before (30 s, left), during (3 s, middle), and immediately after the looming stimulus (10 s, right) in panel (**D**). Max speed, **P = 0.0028; Latency, **P = 0.0028; Time in shelter, **P = 0.0014. Two-tailed *t* test in panel **E**. Two-way repeated-measures ANOVA and Sidak's multiple comparisons test for panel **C**. Data are presented as the mean ± SEM.

To determine whether the ZIr^SST^-Re pathway mediates looming stimulus-evoked defensive behaviors, we bilaterally injected a AAV carrying Cre-dependent ChR2, GtACR1 or control EYFP into the rostral ZI of SST-Cre mice (Figs. 5A, 6A, and S10D, E). We found that optogenetic activation of ChR2-expressing axonal terminals in the Re induced immobility in the open field test (videos 8 and 9) and reduced heart rate, but did not affect voluntary movement in the rotarod test (Fig. 5B–F). Meanwhile, optogenetic inhibition of the axon terminals in the rostral ZI-Re pathway by GtACR1 did not affect locomotion in the open field test (Fig. 6B, C). We next examined the effect of optogenetically silencing the ZIr^SST^-Re pathway in the looming stimulus-induced defensive response. We found the mice exhibited reduced maximum speed, increased latency for returning to the shelter, and decreased time spent hiding in the shelter during flight response upon the looming stimulus in the GtACR1 group comparing with the EYFP group (Fig. 6D, E; videos 10 and 11). Together, these data showed that SST-positive neurons in the rostral ZI form functional inhibitory connections with neurons in the Re. The ZIr^SST^-Re pathway was required for the innate defensive behaviors induced by the looming stimulus.

### Upstream Projections to SST-Positive Neurons in the Rostral ZI

The ZI has been considered an information processing node to globally modulate behaviors [15]. Looming stimulus is a visual threatening signal, so we next used a rabies virus (RV)-based viral trans-synaptic tracing system to investigate the possible upstream nucleus that conveyed vision-related information to the SST-positive neurons in the rostral ZI [35]. A mixture of AAV-DIO-TVA-GFP and AAV-DIO-RVG virus was injected into the rostral ZI of SST-Cre mice to label starter cells. After 2 weeks, the RV-EnVA-dG-dsRed virus was injected into the same coordinates to target starter cells. One week following the RV expression, brains were collected to examine the upstream nuclei that projected to SST-positive neurons in the rostral ZI (Fig. S7A, B). We found that SST-positive neurons in the rostral ZI received broad presynaptic inputs from various regions widely across the brain, including the median prefrontal cortex, cingulate cortex, and secondary visual cortex in the cortex, the SC and PAG in the midbrain, the lateral septal nucleus in the striatum, and the lateral hypothalamus (Fig. S7C, D).

The SC receives direct looming stimulus-evoked signals from ganglion cells in the retina [36] and plays a critical role in looming stimulus-induced defensive behaviors [21]. RV-dsRed^-^positive cells retrogradely labeled by SST-positive neurons in the rostral ZI were mainly distributed in the intermediate (IL) and deep layers (DL) of the SC, but very few cells were found in the superficial layers (Fig. S7E). In addition, the RV-dsRed-positive neurons in the SC were immunostained positive for both glutamate (Fig. S7F, G, 50.34% ± 4.019%) and GABA (Fig. S7H, I, 47.89% ± 3.194%).

### Looming Stimulus Activates Neurons in the Superior Colliculus that Project to the Re-projecting SST-Positive Neurons in the Rostral ZI

**Fig. 7:**
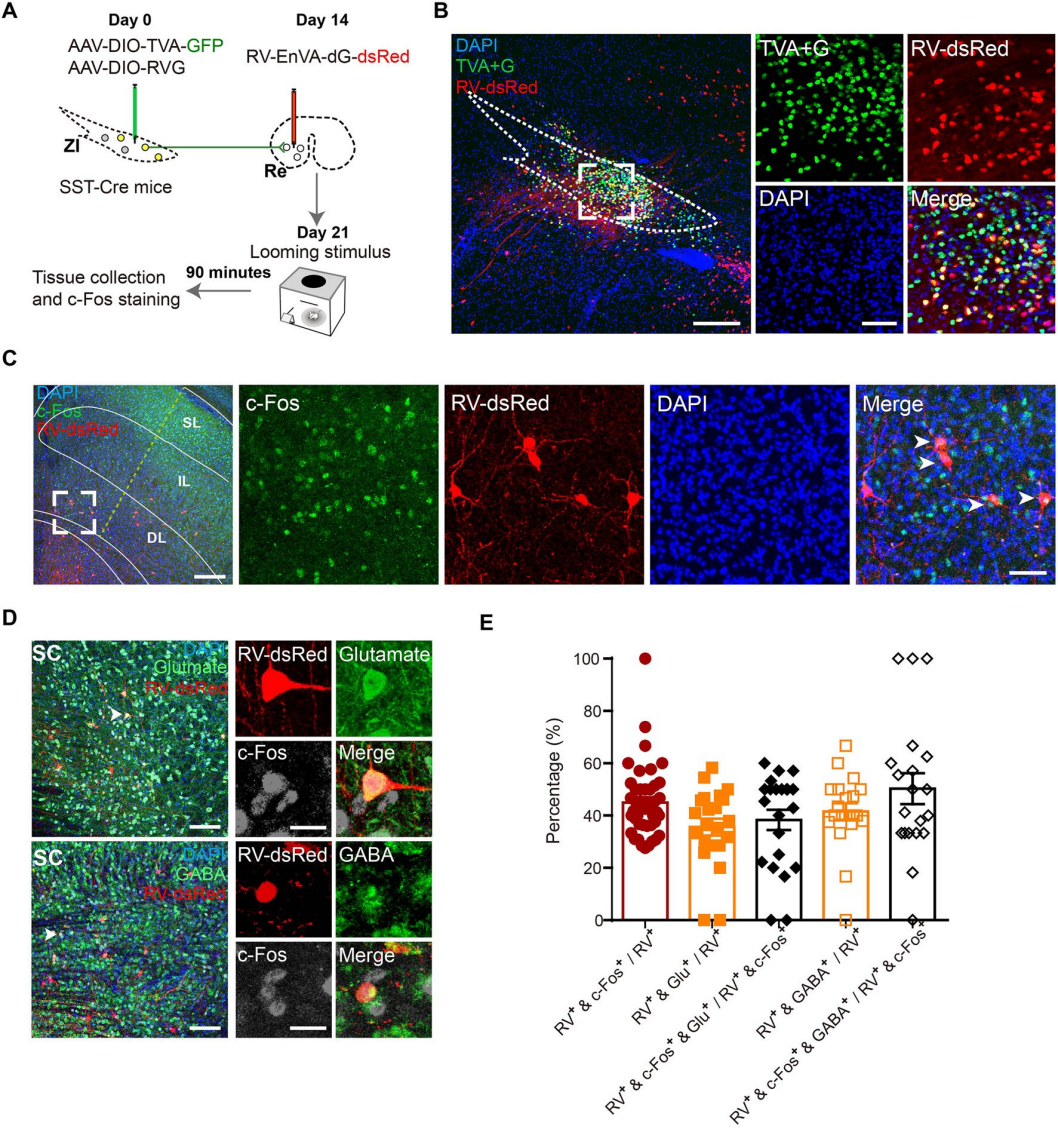
Looming stimulus activates neurons in the superior colliculus (SC) that project to the Re-projecting SST-positive neurons in the rostral ZI. **A** Schematic showing the rabies virus-mediated circuit mapping of upstream projections to the SST-positive neurons in the rostral ZI that project to the Re. The activation of upstream input neurons upon looming stimulus was verified by c-Fos immunostaining. **B** Representative images showing the starter cells in the rostral ZI of SST-Cre mice. Scale bars, 200 μm (left) and 50 μm (right). **C** Representative images of RV-dsRed-labeled neurons in the SC that project to Re-projecting SST-positive neurons in the rostral ZI. Scale bars, 200 μm (left) and 100 μm (right). SL, superficial layers; IL, intermediate layers; and DL, deep layers of the SC. **D** Representative images showing retrograde labeling of neurons in the SC that were co-labeled with glutamate (upper) or GABA (lower). Scale bars, 100 μm (left) and 20 μm (right). **E** Quantification of the proportion of RV-dsRed-labeled neurons that were activated in looming stimulus (RV^**+**^ and c-Fos^**+**^) in total RV-dsRed-labeled neurons (RV^**+**^) in the SC (*n* = 42 slices from 3 mice); The proportion of RV-dsRed-labeled neurons co-labeled with glutamate (RV^**+**^ and Glu^**+**^) (*n* = 22 slices from 3 mice) or GABA (RV^**+**^ and GABA^**+**^) (*n* = 20 slices from 3 mice) in total RV-dsRed-labeled neurons in the SC; The proportion of looming stimulus activated RV-dsRed-labeled neurons co-labeled with c-Fos co-express glutamate (RV^**+**^, c-Fos^**+**^, and Glu^**+**^) or GABA (RV^**+**^**,** c-Fos^**+**^**,** and GABA^**+**^) in total looming stimulus activated RV-dsRed-labeled neurons (RV^+^ and c-Fos^+^) in the SC. Data are presented as the mean ± SEM.

To further confirm whether the Re-projecting SST-positive neurons in the rostral ZI received synaptic inputs from the SC, we injected a mixture of AAV-DIO-TVA-GFP and AAV-DIO-RVG into the rostral ZI of SST-Cre mice. After 2 weeks, we injected RV-EnVA-dG-dsRed into the Re of the same mice to retrogradely label upstream inputs into the Re-projecting SST-positive neurons in the rostral ZI. Mice were further exposed to the looming stimulus-evoked flight behavioral tests to determine which upstream nuclei were activated (Fig. 7A, B). We found that the looming stimulus induced c-Fos expression, a marker of recent neuronal activation, in RV-dsRed-positive cells in the IL and DL of the SC (Fig. 7C, E, 44.92% ± 2.134%). In addition, the RV-dsRed-positive cells in the SC were immunostained positive for both glutamate (Fig. 7D, E, 35.59% ± 3.197%) and GABA (Fig. 7D, E, 41.53% ± 3.199%). And looming-activated RV-dsRed-positive cells in the SC were also immunostained positive for both glutamate (Fig. 7D, E, 38.32% ± 3.885%) and GABA (Fig. 7D, E, 50.29% ± 5.925%). These results suggested that neurons in the SC projecting to the Re-projecting SST-positive neurons in the rostral ZI (SC-ZIr^SST^-Re pathway) were activated by the looming stimulus. Together, our data revealed a tri-synaptic inter-regional circuit from the SC to Re-projecting SST-positive neurons in the rostral ZI. The SC-ZIr^SST^-Re tri-synaptic circuit participates in the looming stimulus-evoked defensive behaviors.

## Discussion

By integrating viral-mediated tracing, immunohistochemistry, electrophysiology, *in vivo* fiber photometry, and optogenetic and pharmacological stimulation, we demonstrated that SST-positive neurons in the rostral ZI participate in the defensive behavior induced by a looming stimulus. Subsequently, we revealed that these SST-positive neurons in the rostral ZI established functional GABAergic projections to the glutamatergic neurons in the Re. The ZIr^SST^-Re pathway mediated looming stimulus-induced defensive behavior. Finally, we identified a tri-synaptic inter-regional circuit from the SC to Re-projecting SST-positive neurons in the rostral ZI that might be involved in the transmission of visual threat by a looming stimulus to elicit innate fear defensive behavior.

We found that SST-positive neurons in the rostral ZI were activated upon the onset of a looming stimulus. Previous studies have reported changes in heart rate, blood pressure, and breathing caused by manipulation of the ZI. For example, Loewy *et al.* showed that microinjection of L-glutamate into ZI in rats reduces blood pressure and heart rate [37]. Similarly, Bohus *et al.* found that low-intensity electrical stimulation of the ZI produced a sustained reduction in heart rate and blood pressure in rats [38]. We recorded a pronounced bradycardia with the optogenetic activation of ChR2-expressing SST-positive neurons in the rostral ZI (Fig. 3H–J), consistent with previous reports of altered cardiovascular and autonomic physiological responses when stimulating ZI neurons [37, 38]. Bradycardia is often an important feature of the freezing response to threat [17, 39], therefore, the immobility induced by optogenetic activation of SST-positive neurons in the rostral ZI might represent freezing-like behavior induced by innate fear stimulus.

GABAergic projections from the rostral ZI to the PAG are involved in the gain modulation of noise-induced flight behavior [8]. Indeed, we found sparse projections from SST-positive neurons in the rostral ZI to the dorsal-medial and lateral-ventral PAG, but very few innervated the dorsal-lateral PAG (Fig. S8). Intriguingly, we identified extensive GABAergic axon projections of SST-positive neurons in the rostral ZI to the Re. The excitatory neurons in the Re project to the mPFC to promote saliency-enhancing reactions to looming visual threats, which exhibited more tail rattling, a confrontational response to threats in rodents [23]. Therefore, optogenetic activation of the ZIr^SST^-Re GABAergic pathway might suppress the projections from Re to the mPFC, thus promoting saliency-reducing passive defensive responses such as freezing to an overhead looming stimulus (Figs. 4 and 5).

Excitatory neurons in the SC relay visual information to downstream brain regions such as the lateral posterior thalamic nucleus [20, 21], parabigeminal nucleus [19, 21], and ventral tegmental area [22] to regulate the flight or freezing behavior of mice under a looming stimulus. Interestingly, our study indicated that SST-positive neurons in the rostral ZI also receive projections from both glutamatergic and GABAergic neurons in the ILs and DLs of the SC (Fig. S7). Moreover, c-Fos results showed that only a fraction of these neurons were directly activated by the looming stimulus (Fig. 7). Thus, our results demonstrated that the SC-ZIr^SST^-Re tri-synaptic circuit participates in the looming stimulus-induced flight behavior. Neurons in the ILSC and DLSC do not receive direct signals from the retina, suggesting that the looming stimulus-related information may be transmitted directly through the intralaminar projection from neurons in the superficial layers of the SC to the neurons in the ILSC and DLSC [40–42]. Previous studies have shown that an excitatory projection from SC to ZI can modulate predatory behavior [4]. The role of GABAergic neurons in the SC that project to SST-positive neurons in the rostral ZI in response to a looming stimulus warrant further investigation.

Previous studies have suggested that a looming stimulus can induce two defensive states in mice, flight or freezing [18, 43]. However, the vast majority of mice demonstrated flight, but not freezing, in the looming paradigm we used, presumably related to the brightness of the surrounding environment or the number of looming stimulus cycles used in our experiments. Therefore, we were unable to determine the activity of SST-positive neurons in the rostral ZI in the freezing state induced by a looming stimulus. We also recorded an increase of the GCaMP6s fluorescence intensity in SST-positive neurons in the rostral ZI elicited by a sweeping stimulus, which induced freezing but not flight defensive behavior in mice (Fig. S2A).

In response to both looming and sweeping stimuli, we recorded increased Ca^2+^ signals of population activity in SST-positive neurons in the rostral ZI using fiber photometry. In addition, we found optogenetically silencing SST-positive neurons in the rostral ZI blocked the flight response under a looming stimulus or the freezing response under a sweeping stimulus (Figs. 2 and S5). However, optogenetically activating SST-positive neurons in the rostral ZI induced freezing-like behavior (Fig. 3E–J). We hypothesize that these SST-positive neurons in the rostral ZI might be composed of functionally distinct subtypes that project to different target areas such as the Re, PAG, or precuneiform area (PrCnF) (Figs. 4 and S8). In addition, different neuronal subtypes or sub-regions of the PAG are responsible for different physiological functions in active and passive defensive behavior [44]. Thus, different projections from SST-positive neurons in the rostral ZI might mediate distinct behavioral responses. We found the strongest axonal projections from SST-positive neurons in the rostral ZI to the Re, while fewer axonal projections were distributed in PAG or PrCnF (Figs. 4 and S8). Therefore, optogenetic activation of SST-positive neurons in the rostral ZI induced freezing-like responses that were mediated by Re-projecting SST-positive neurons (Figs. 4 and 5). Future experiments are warranted to reveal the cell type-specific projections in the rostral ZI that mediate flight responses.

Recently, Kelly Tan's lab reported the involvement of SST-positive neurons in the ZI in the regulation of anxiety behavior [12]. They found that the SST-positive neurons in the ZI are activated when mice are in anxiety-inducing environment. In the EPM test, optogenetic activation of SST-positive neurons reduced the time spent in the open arms and the number of times the mice entered the open arms, but optogenetic inhibition of SST-positive neurons in the ZI did not affect the performance of the mice in EPM test. Similarly, we also found that optogenetic activation of SST-positive neurons in the rostral ZI resulted in reduced time spent in the open arm in the EPM test (Fig. S9). However, we reasoned that decrease of time in the open arm might be an accompanying behavior of freezing-like response when optogenetically activating SST-positive neurons in the rostral ZI (Fig. 3).

In summary, our study delineated an inter-regional tri-synaptic circuit from the SC to Re-projecting SST-positive neurons in the rostral ZI that mediated the innate fear response. We further demonstrated that the SC-ZIr^SST^-Re tri-synaptic circuit participated in the looming stimulus-evoked defensive behaviors. These results further enrich the understanding of the functional projection map of ZI neuron subtypes that are essential components of innate fear circuitry in the brain.

## Supplementary Information

Below is the link to the electronic supplementary material.

Supplementary file1 (PDF 1776 KB)

Supplementary file2 (RAR 21294 KB)
