# Supplementary material for: Somatostatin-Positive Neurons in the Rostral Zona Incerta Modulate Innate Fear-Induced Defensive Response in Mice
Source: Neurosci Bull. 2022 Oct 19;39(2):245–60. doi: 10.1007/s12264-022-00958-y (PMC9905479; doi:10.1007/s12264-022-00958-y)
Supplement: Supplementary file 1 — Supplementary file1 (PDF 1776 KB) [file 12264_2022_958_MOESM1_ESM.pdf]

Supplemental Materials

Supplemental Figures and Figure Legends

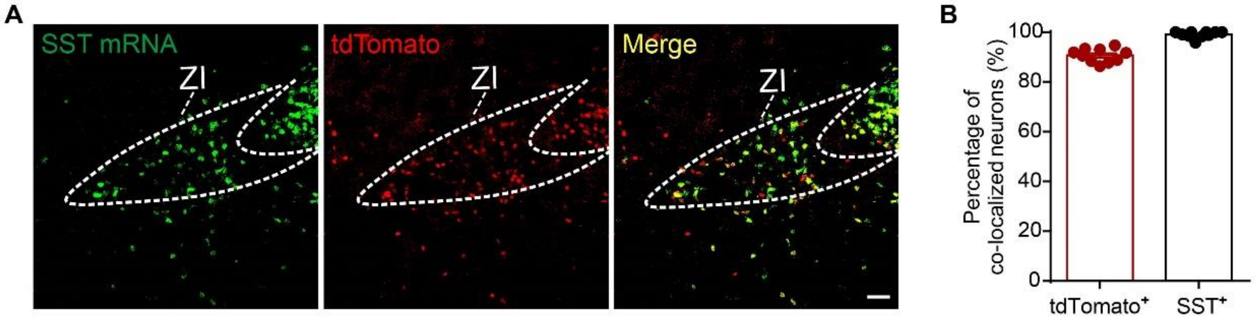

Fig. S1 SST mRNA is strongly expressed in SST-positive neurons in the rostral ZI. **A** Representative images showing the co-expression of SST mRNA and tdTomato-positive neurons in the rostral ZI in brain slice from SST x Ai14 mice. Scale bar, 50 μm. **B** The percentages of SST mRNA expression (SST^+) in tdTomato-positive ($tdTomato^+$) neurons in the rostral ZI (n = 10 slices from 3 mice). Data are presented as mean ± SEM.

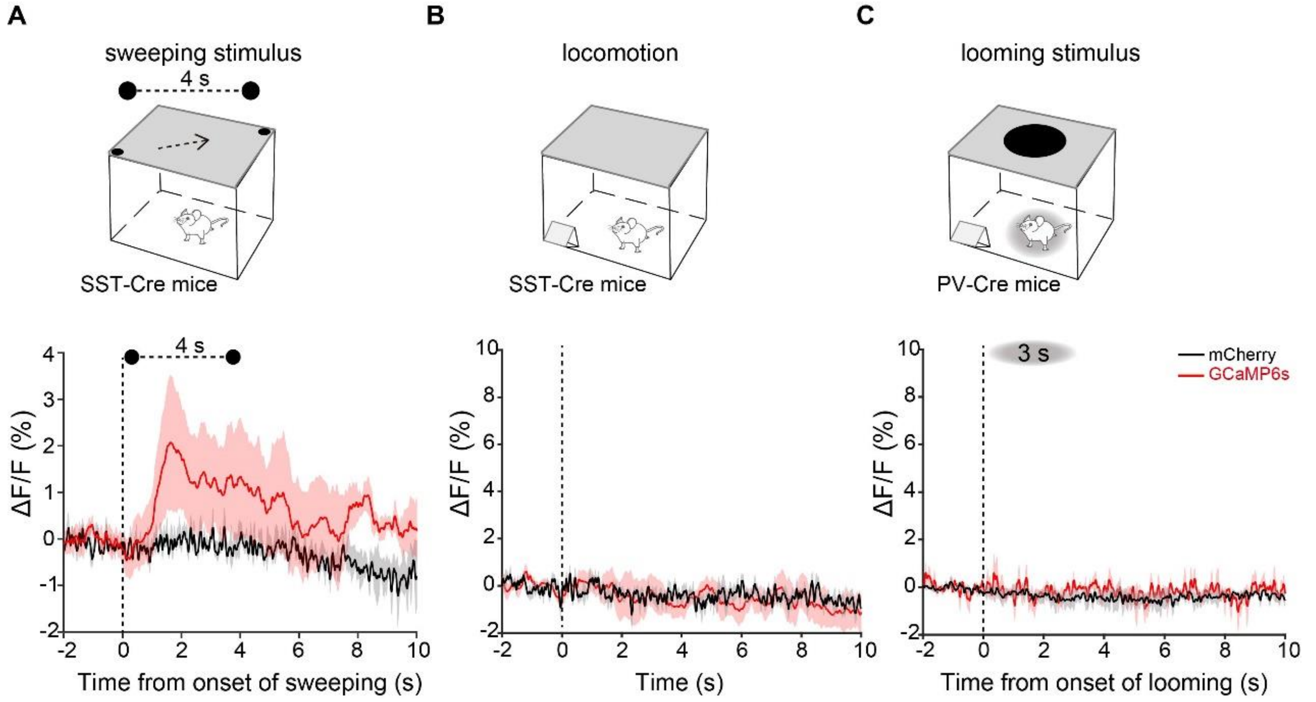

Fig. S2 SST-positive neurons in the rostral ZI are activated in the innate defensive response induced by an exteroceptive stimulus. **A** Schematic paradigm and average change of calcium fluorescent intensity of SST-positive neurons in the rostral ZI in free-moving SST-Cre mice during sweeping stimulus. **B** Schematic paradigm and average change of calcium fluorescent intensity of SST-positive neurons in the rostral ZI in free-moving SST-Cre mice. **C** Schematic paradigm and average change of calcium fluorescent intensity of PV-positive neurons in the rostral ZI in free-moving PV-Cre mice during looming stimulus. The gray dotted line indicates the onset of sweeping or looming stimulus

(red line, GCaMP6s virus channel recording; black line, mCherry control virus channel recording. Shaded areas around means indicate SEM).

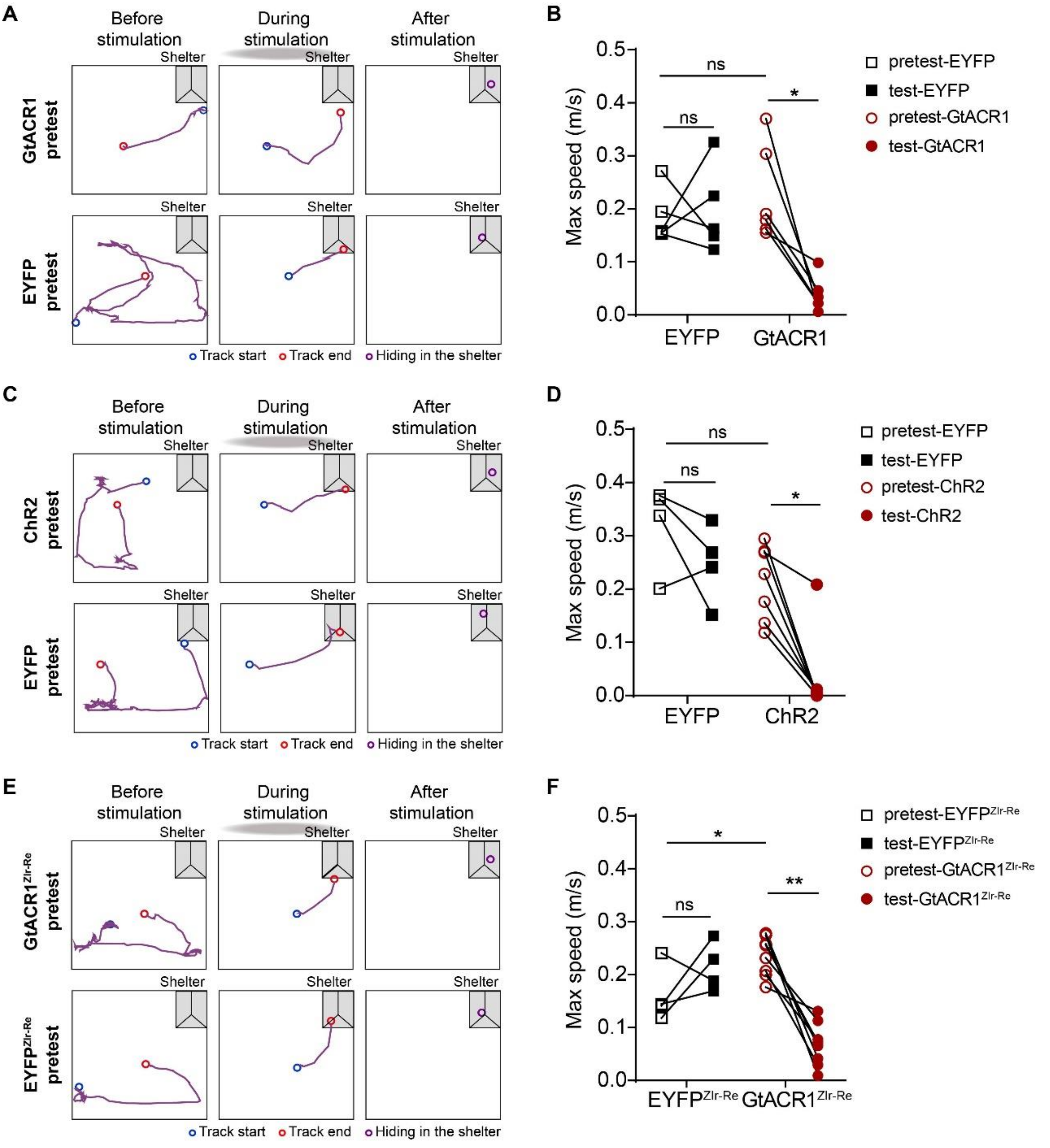

Fig. S3 Representative traces and statistical analysis of mouse movement in the pre-test. Representative traces **(A, C** and **E)** and statistical analysis **(B, D** and **F)** of animal movement in GtACR1-group, ChR2-group, GtACR1$^{\mathrm{ZIr\text{-}Re}}$-group (upper row in **A, C** and **E**) and EYFP-group (lower row in **A, C** and **E**) in pre-test. Max speed, *P = 0.0313 in **B**; *P = 0.0156 in **D**; **P = 0.0078 in **F**; two-tailed paired t test. *P = 0.0485 in **F**, two-tailed unpaired t test. Data are presented as mean ± SEM.

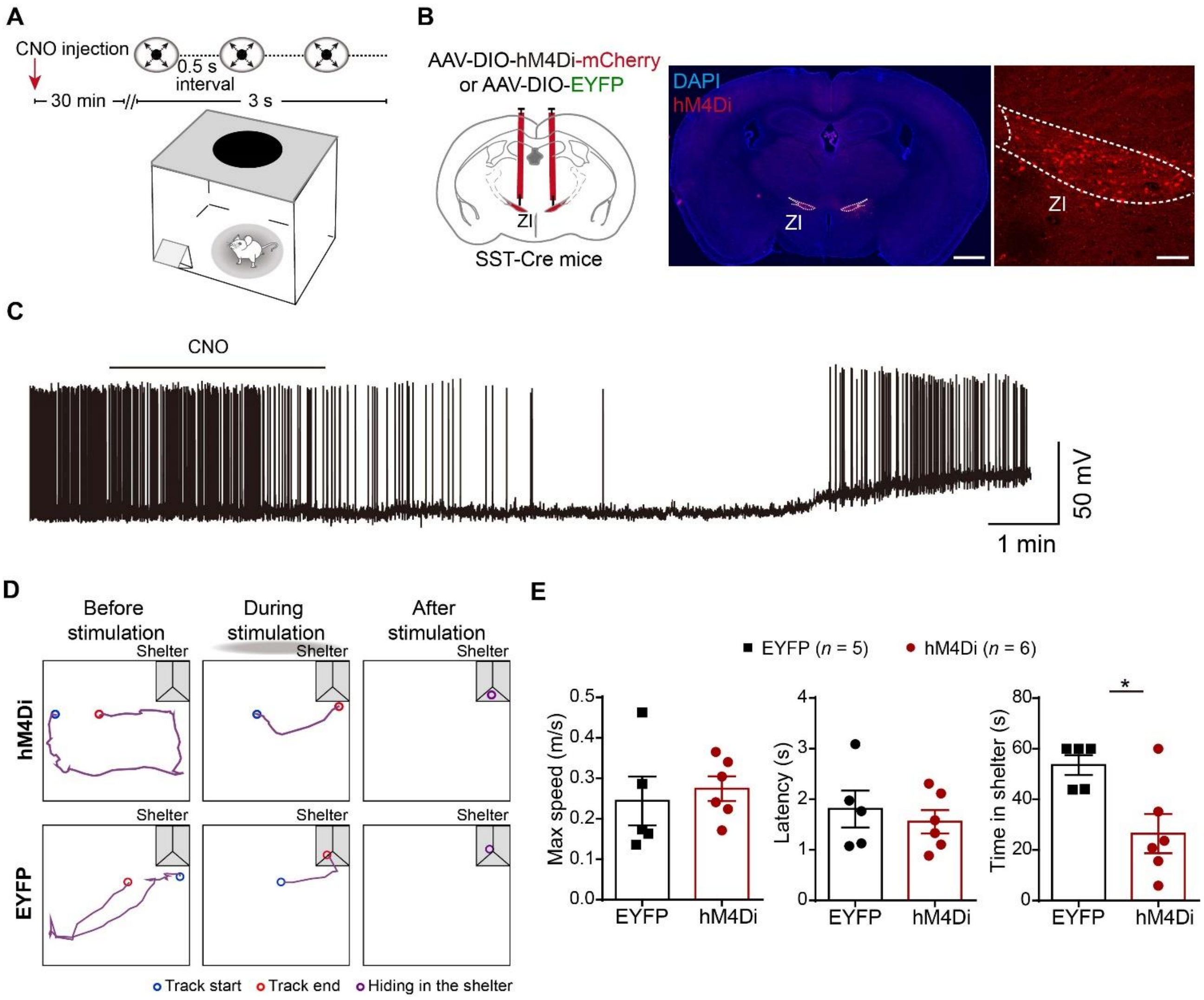

Fig. S4 Pharmacological inhibition of SST-positive neurons in the rostral ZI partially affects flight responses to a looming stimulus. **A** Schematic diagram showing bilateral labeling Cre-dependent AAV mediated hM4Di-mCherry or EYFP expression in SST-positive neurons in the rostral ZI. CNO was applied 30 min before the onset of looming stimulus. **B** Representative images showing hM4Di expression in the rostral ZI in SST-Cre mice. Scale bars, 1mm and 100 μm (zoom in area), respectively. **C** Representative whole-cell recording traces for an example hM4D(Gi)-expressing neuron before and after CNO perfusion. After 1 min baseline recording, CNO (5 μM) in ACSF were bath applied and then washed out with ACSF. Scale bar, 50 mV. **D** Representative traces and statistical analysis (**E**) of hM4Di-group (upper row) and EYFP-group (lower row) movement before looming stimulus (30 s, left), during looming stimulus (3 s, middle) and immediately after looming stimulus (10 s, right). Max speed, $P = 0.4156$; latency, $P = 0.7706$; time in shelter, $*P = 0.0303$; two-tailed unpaired t test.

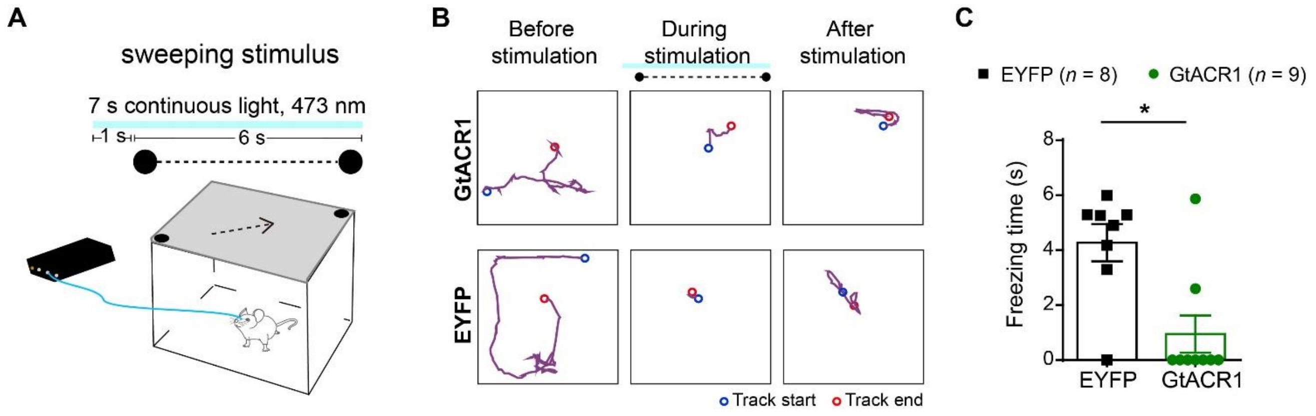

Fig. S5 Optogenetic inhibition of SST-positive neurons in the rostral ZI attenuates defensive behaviors evoked by a sweeping stimulus. **A** Schematic diagram of optogenetic manipulation of bilateral rostral ZI SST-positive neurons during the sweeping stimulus. Continuous blue light stimulation was delivered through optic fibers 1 s before the onset of sweeping stimulus and last for 7 s. **B** Representative traces of GtACR1-group (upper row) and EYFP-group (lower row) movement before sweeping stimulus (30 s, left), during sweeping stimulus (6 s, middle) and immediately after sweeping stimulus (10 s, right). **C** Statistical analysis of the freezing time during the sweeping stimulus and optogenetic stimulation in GtACR1-group compared with EYFP group. Freezing time, $*P = 0.0106$, two-tailed unpaired *t* test.

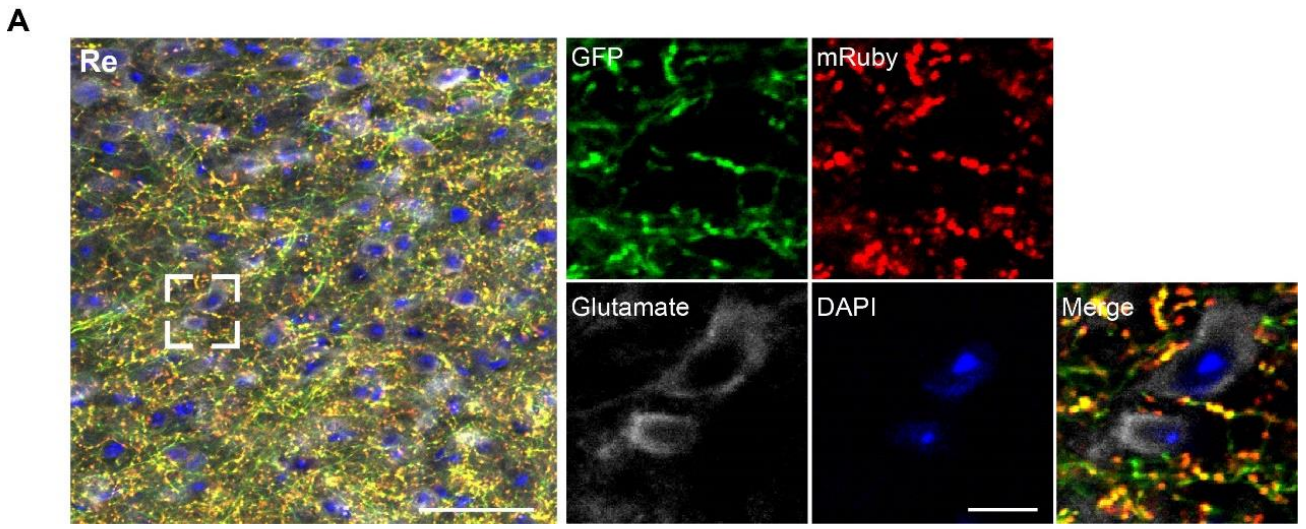

Fig. S6 Glutamatergic neurons in the Re receive projections from SST-positive neurons in the rostral ZI. **A** Representative images showing neurons in Re that received projection from SST-positive neurons in the rostral ZI which co-labeled with glutamate. Scale bars, 50 μm and 10 μm (zoom in area), respectively.

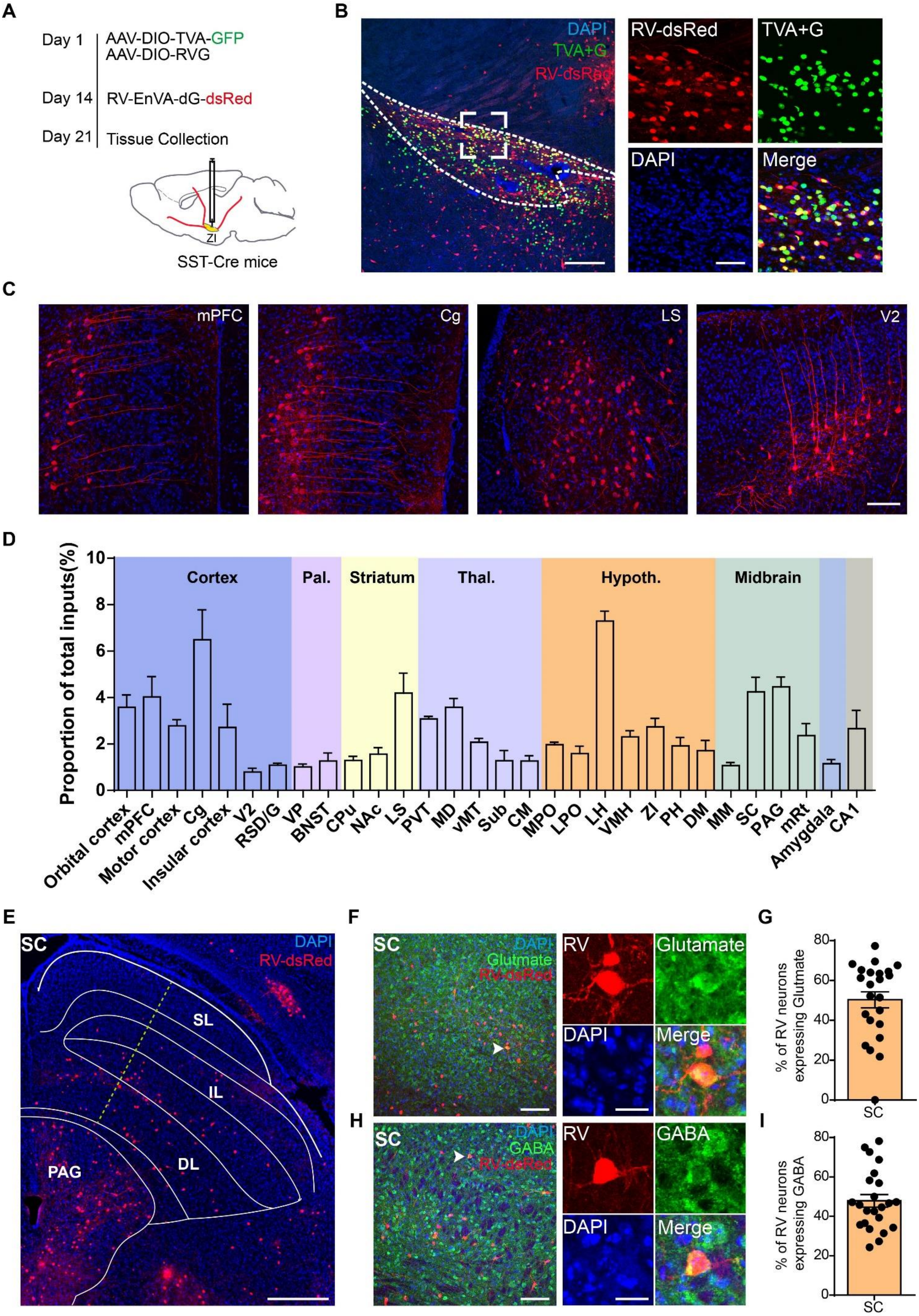

Fig. S7 Retrograde tracing of the upstream projections to SST-positive neurons in the rostral ZI. **A**

Rabies virus-based cell type-specific monosynaptic tracing strategy. **B** Representative images showing the starter cells in the rostral ZI of SST-Cre mice. Starter cells were double-labeled with TVA+G (green) and RVdG (red), and therefore are yellow. Scale bars, 200 μm and 50 μm (zoom in area), respectively. **C** Representative images showing distribution of presynaptic patterns in some brain areas with inputs to SST-positive neurons in the rostral ZI. Scale bar, 100 μm. **D** Quantitative analysis of whole-brain inputs to SST-positive neurons in the rostral ZI, shown as proportion of total number of cells counted that are located in one region. **E** Representative image showing distribution of RV labeled presynaptic neurons in the SC with inputs to SST-positive neurons in the rostral ZI. Scale bar, 1 mm. SL, superficial layer of SC; IL, intermediate layer of SC; DL, deep layer of SC. **F-I** Representative images and quantification showing retrograde labeling neurons which co-labeled with glutamate **(F, G)** (*n* = 23 slices from 3 mice) or GABA **(H, I)** (*n* = 23 slices from 3 mice) in the SC. Scale bars, 100 μm and 20 μm (zoom in area), respectively.

Abbreviations: Pal., pallidum; Hypoth., hypothalamus; Thal., thalamus; mPFC, medial prefrontal cortex; Cg, cingulate cortex;V2, secondary visual cortex; RSD/G, retrosplenial granular cortex; VP, ventral pallidum; BNST, bed nucleus of the stria terminalis; CPu, caudate putamen; NAc, nucleus accumbens; LS, lateral septal nucleus; PVT, paraventricular thalamic nucleus; MD, mediodorsal thalamic nucleus; vMT, ventral midline thalamus; Sub, submedius thalamic nucleus; CM, central medial thalamic nucleus; MPO, medial preoptic nucleus; LPO, lateral preoptic nucleus; LH, lateral hypothalamus; VMH, ventromedial hypothalamic nucleus; ZI, zona incerta; PH, posterior hypothalamic nucleus; DM, dorsomedial hypothalamic nucleus; MM, medial mammillary nucleus; SC, superior colliculus; PAG, periaqueductal gray; mRt, mesencephalic reticular formation; CA1, field CA1 of the hippocampus.

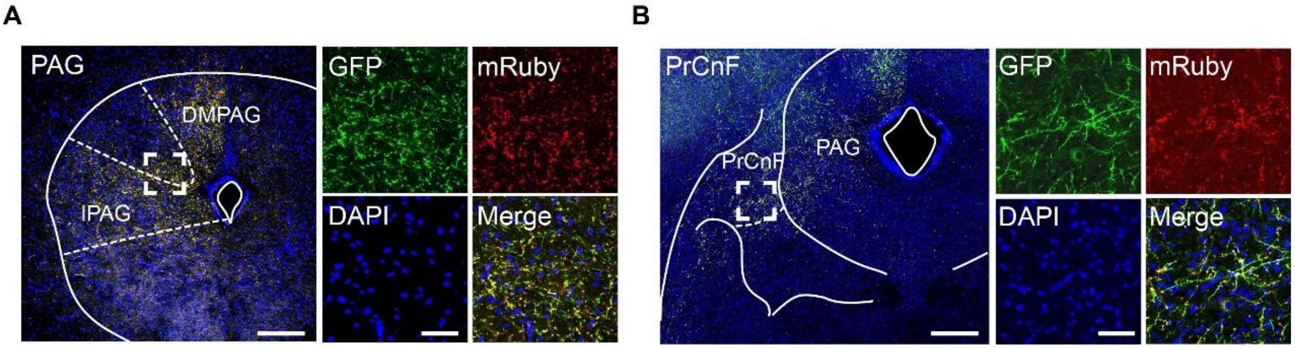

Fig. S8 Anterograde tracing from SST-positive neurons in the rostral ZI. **A** Representative images showing axon terminals expression of mGFP-mRuby in the PAG and precuneiform area (PrCnF) **(B)** from SST-positive neurons in the rostral ZI. Scale bars, 200 μm and 50 μm (zoom in area), respectively.

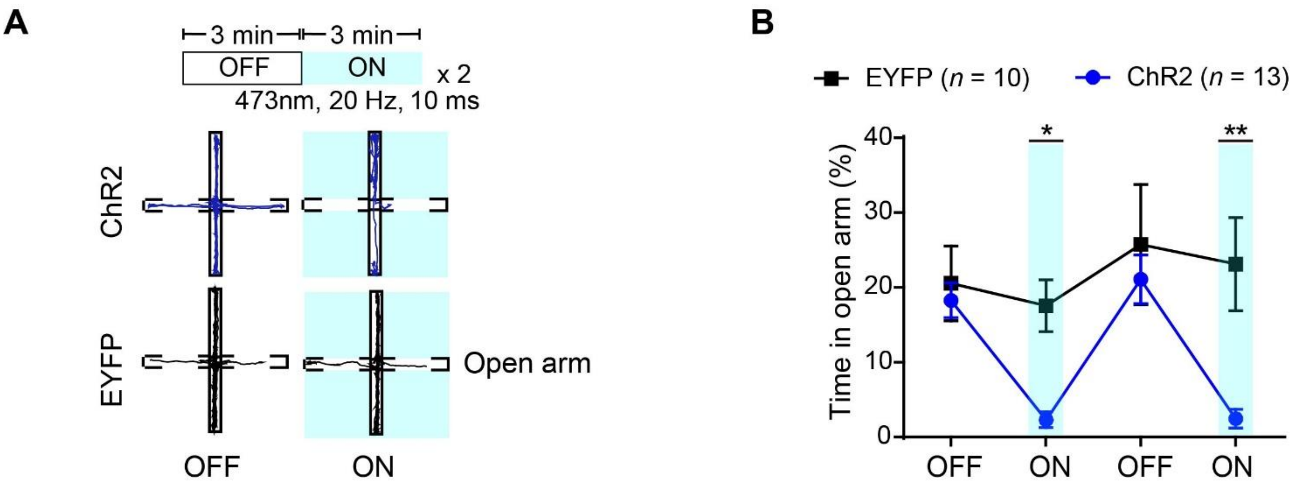

Fig. S9 Optogenetic activation of SST-positive neurons in the rostral ZI induce anxiety-like behavior. **A** Representative traces and statistical analysis (**B**) showing the movement of mice in elevated plus maze (EPM) test upon light stimulation of ChR2-group (upper row in **A**) and EYFP-group (lower row in **A**), respectively. Time in open arm (**B**), $F_{(3,66)} = 4.679$, $P = 0.0050$. *$P = 0.0299$, **$P = 0.0015$ for laser on stage comparison. Two-way repeated-measures ANOVA and Sidak's multiple comparisons test. Data are presented as mean ± SEM.

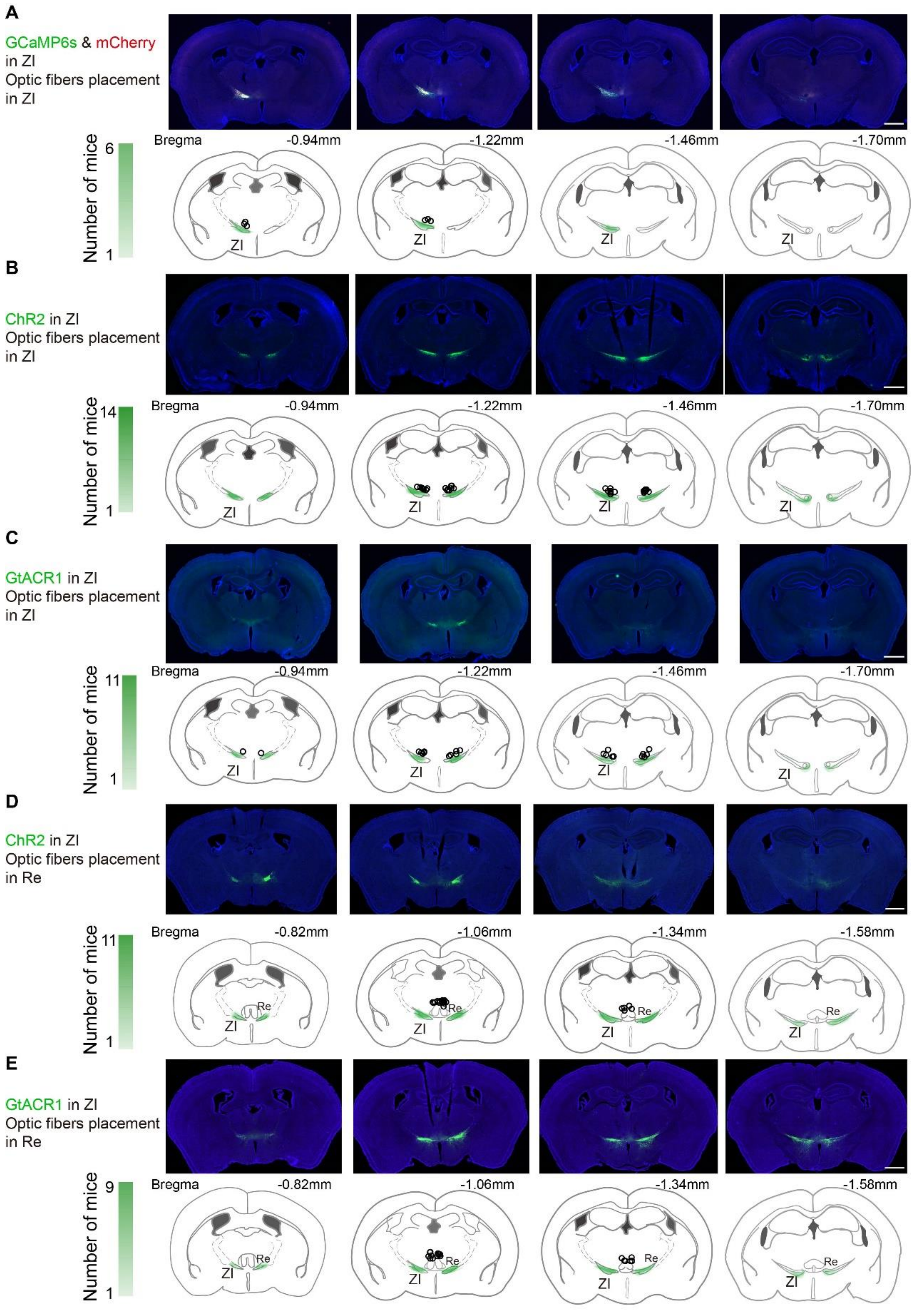

Fig. S10 Verification of virus expression and optical fiber placement. **A-E** Representative images

showing anterior-to-posterior brain slices from one mouse in which virus was injected into the rostral ZI (upper row) and overlay of different virus expression in the rostral ZI and optic fibers placement in the rostral ZI or Re in mice used for fiber photometry and behavior tests (lower row). Scale bar, 1 mm.

Videos

video 1 Looming test

video 2 Optogenetic inhibition of ZIr SST neurons-looming-GtACR1

video 3 Optogenetic inhibition of ZIr SST neurons-looming-EYFP

video 4 Optogenetic activation of ZIr SST neurons-looming-ChR2

video 5 Optogenetic activation of ZIr SST neurons-looming-EYFP

video 6 Optogenetic activation of ZI SST neurons-OFT-ChR2

video 7 Optogenetic activation of ZI SST neurons-OFT-EYFP

video 8 Optogenetic activation of ZIr-Re-OFT-ChR2

video 9 Optogenetic activation of ZIr-Re-OFT-EYFP

video 10 Optogenetic inhibition of ZIr-Re-looming-GtACR1

video 11 Optogenetic inhibition of ZIr-Re-looming-EYFP
